# Network Neuroscience of Human Multitasking: Local Connections Matter

**DOI:** 10.1002/hbm.70434

**Published:** 2025-12-15

**Authors:** Marie Mueckstein, Kirsten Hilger, Stephan Heinzel, Urs Granacher, Michael A. Rapp, Christine Stelzel

**Affiliations:** ^1^ International Psychoanalytic University Berlin Germany; ^2^ University of Potsdam Potsdam Germany; ^3^ University of Würzburg Würzburg Germany; ^4^ Freie Universität Berlin Berlin Germany; ^5^ TU Dortmund University Dortmund Germany; ^6^ University of Freiburg Freiburg im Breisgau Germany

**Keywords:** crosstalk, dual task, executive control, functional connectivity, modality compatibility

## Abstract

The neural basis of multitasking costs is subject to continuing debate. Cognitive theories assume that the overlap of task representations may lead to between‐task crosstalk in concurrent task processing and thus requires cognitive control. Recent research suggests that modality‐based crosstalk contributes to multitasking costs, involving central overlap of modality‐specific representations. Consistently increased costs for specific modality pairings (visual‐vocal and auditory‐manual vs. visual‐manual and auditory‐vocal) were demonstrated (modality‐compatibility effect), which were recently linked to representational overlap in the auditory cortex. However, it remains unclear whether modality‐based crosstalk emerges from overlapping patterns of global brain connectivity and whether resolving it requires additional involvement of cognitive control as reflected in the fronto‐parietal control network. This preregistered functional imaging study investigates these questions in 64 healthy, young human adults. Specifically, we focus on the modality‐compatibility effect in multitasking by employing functional connectivity (FC) analysis. First, we tested the FC similarity FC dissimilarity between the single‐task networks. Second, we compared the strength of the control network in whole‐brain FC between dual tasks. We found no evidence for differences in FC dissimilarities of single‐task networks between modality pairings and no additional involvement of the control network during dual tasks by comparing the global connectivity. However, unregistered post hoc connectivity analysis revealed the first evidence for a correlation of the (behavioral) modality‐compatibility effect with local FC. This effect was locally restricted to FC between lateral frontal and sensory auditory regions, consistent with the modality‐based crosstalk assumption. More generally, the findings suggest that robust behavioral differences in multitasking are not necessarily related to global functional connectivity differences but might be related to functionally specific local connectivity changes.

## Graphical abstract

Multitasking limitations can be related to how our senses interact in the brain. This study shows that performance costs in multitasking depend on local brain connections—especially between hearing and control areas—rather than broad brain network differences, supporting the idea that sensory overlap drives multitasking difficulties.

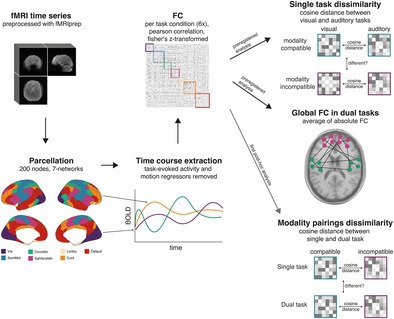

## 1 Introduction

Whether it is cooking while listening to a podcast or driving while talking on the phone—multitasking is omnipresent in everyday life, even though it usually comes with performance costs. Neuroimaging studies investigating the neural basis of performing two tasks concurrently (i.e., dual‐tasking) show consistent activity in frontoparietal regions associated with cognitive control (*see* meta‐analysis by Worringer et al. 2019). This activity occurs when comparing dual‐task to single‐task activity and dual tasks with different cognitive demands. Cognitive control may also be involved in processing representational overlap between tasks in a dual‐task context. Recently, several functional imaging (fMRI) studies (Alavash et al. 2015; Garner and Dux 2015; Mueckstein et al. 2025; Nijboer et al. 2014; Paas Oliveros et al. 2023) complemented the available behavioral research on the role of representational overlap for dual‐task costs (Janczyk et al. 2014; Koch 2009). A widespread assumption is that representational overlap causes central crosstalk, that is, the unintended exchange of information between tasks, thus negatively affecting performance (Lien and Proctor 2002; Logan and Gordon 2001; Navon and Miller 1987).

**FIGURE 1:**
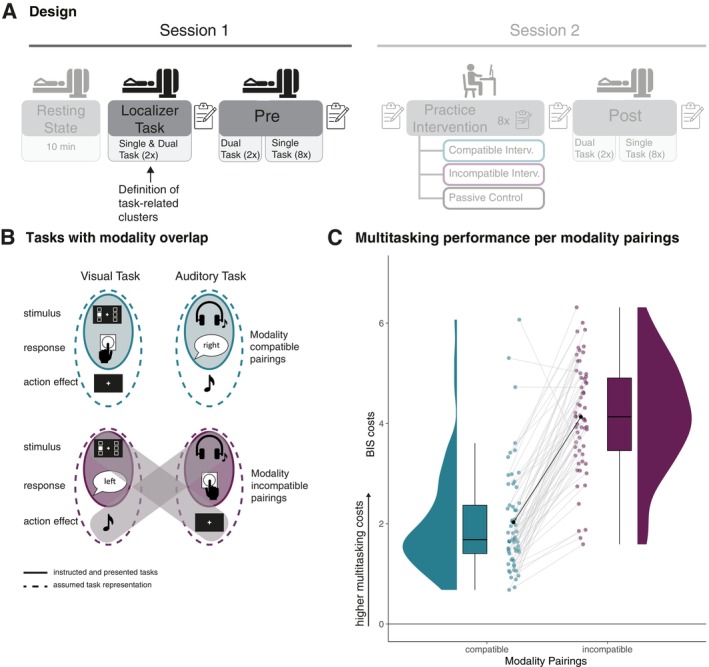
Experimental design, tasks and behavioral results. (A) Overview of the two fMRI sessions. The parts of the study that are not relevant here are hidden and will be reported elsewhere. Localizer tasks are the same tasks as used in the Pre part in both modality pairings and for single and dual tasks in a block‐design (two runs). This data is used to define the univariate task‐activity clusters. The main part (Pre) consists of two dual‐task runs, one per modality pairing, followed by eight single‐task runs, each containing eight blocks, one for each single‐task type (task complexity x modality pairing). (B) The upper part of the figure depicts the stimulus–response pairings for the modality‐compatible pairing, comprising a visual stimulus with a manual response and an auditory stimulus combined with a vocal response. For each response, the corresponding natural action effects are depicted as well. Note that the action effect of the manual response is not exclusively visual but also somatosensory. Likewise, the action effect of the vocal response is typically auditory (i.e., hearing oneself speaking) but also somatosensory (i.e., feeling one's mouth move). The lower part depicts the modality‐incompatible pairing. The visual stimulus is paired with a vocal response and the auditory stimulus with a manual response. In this condition, the match between action‐effect modality and stimulus modality is between tasks, potentially causing interference when presented as a dual task, due to higher overlap. (C) The graph provides the distribution, boxplot, mean, and individual performance per modality pairing of the balanced integrated score (BIS) cost parameter. Multitasking cost is the difference between dual task and single tasks, measured as BIS, which is an integration of reaction times and accuracies (BIS = 0 means no difference between single and dual task performance). The graph demonstrates the robustly higher multitasking cost for the modality‐incompatible pairing, compared to the modality‐compatible pairing.

Recently, Mueckstein et al. (2025) investigated the neural basis of modality‐based crosstalk—a specific type of crosstalk, where central overlap between modality‐specific information is assumed to cause dual‐task costs. Several behavioral studies provided evidence for robustly increased dual‐task costs when pairing visual‐vocal (VV) with auditory‐manual (AM) tasks (i.e., modality‐incompatible) compared to visual‐manual (VM) with auditory‐vocal (AV) tasks (i.e., modality‐compatible) (Göthe et al. 2016; Hazeltine et al. 2006; Mueckstein et al. 2022; Stelzel et al. 2006). According to the modality‐based crosstalk account, this modality‐compatibility effect in dual tasks arises from the overlap between the stimulus modality in one task (e.g., auditory stimulus) and the anticipated sensory action consequence (e.g., auditory action effect of a vocal response) in the simultaneously performed tasks (Figure 1B) (Schacherer and Hazeltine 2020, 2021, 2023).

Mueckstein et al. (2025) provided neural evidence for this hypothesis by applying multi‐voxel pattern analysis to fMRI data. Between‐task overlap in modality‐incompatible single tasks was exclusively present in the auditory cortex, supporting the role of local modality‐specific representational overlap for dual‐task performance. Additionally, a region‐of‐interest (ROI) analysis on univariate fMRI data revealed greater involvement of the left inferior frontal sulcus during the performance of modality‐incompatible compared to modality‐compatible dual tasks, suggesting higher cognitive control demands for modality‐incompatible pairings (Stelzel et al. 2006).

However, the human brain is more than a collection of circumscribed regions operating in isolation. Network neuroscience highlights the importance of dynamic interactions (Rubinov and Sporns 2010) and stresses the importance of considering whole‐brain connectivity (Sporns 2011). Yet, little is known about these interactions in dual‐task processing, including the processing of modality‐based crosstalk. Our study closes this gap. First, we compared whole‐brain single‐task functional connectivity (FC) patterns between modality pairings. Alavash et al. (2015) demonstrated that greater overlap between single‐task modules predicts higher dual‐task costs. Combining this finding with the results of Mueckstein et al. (2025), we hypothesized that the single‐task FC pattern overlaps more strongly (lower dissimilarity) for modality‐incompatible pairings than for modality‐compatible pairings. Second, we compared dual‐task‐related FC patterns between modality pairings to investigate whether the strength of connectivity of fronto‐parietal regions associated with cognitive control processes in dual tasks (Worringer et al. 2019) differ. Based on the previous univariate data (Stelzel et al. 2006), we hypothesized larger involvement of the control network (Yeo et al. 2011) in modality‐incompatible dual tasks related to resolving modality‐based crosstalk.

We observed no significant differences in FC between modality pairings, neither between the single‐task similarity of FC patterns nor in the involvement of the control network between dual tasks. However, unregistered post hoc analyses revealed higher local FC between lateral frontal regions and auditory regions in the modality‐incompatible compared to the modality‐compatible dual task. This difference in FC was associated with individual differences in dual‐task performance. Our results thus provide first evidence for the assumption that controlling the representational overlap of modality‐specific information by engaging lateral frontal brain regions is relevant for successfully resolving modality‐based crosstalk in multitasking.

## 2 Methods

### 2.1 Preregistration

This study is part of a larger research project (see Figure 1A). The project was pre‐registered before any data analysis took place (https://osf.io/whpz8) and contains two different methodological approaches using the same sample. The first was a multi‐voxel pattern analysis (MVPA) reported in Mueckstein et al. (2025), and the second is reported here. Accordingly, sections in the methods are mostly copied from the preregistration and shortened. Note that any deviations from the preregistration are explicitly marked as such.

### 2.2 Participants

In total, 71 healthy, right‐handed adults aged 18–30 with German as their first language (or comparable level) and normal or corrected‐to‐normal vision participated in the study. We recruited participants via flyers and mailing lists of local universities and online advertisements. Exclusion criteria were any neurological or psychiatric diseases, current medical conditions that could potentially influence brain functions, past or present substance abuse (alcohol and drugs), a self‐reported weakness in distinguishing left and right, and common contraindications for MRI scanning. Table 1 provides the final sample size along with age and gender distributions per analysis. We provide a detailed overview of why participants were excluded and how many participants were part of both analyses (MVPA and connectivity) in the Supporting Information (Table S1). The specific criteria for exclusion are reported in the sections “Behavioral tasks” and “Preprocessing functional brain network connectivity.” All participants provided written informed consent before the study started and were reimbursed with 60 € or course credit. The ethics committee of the Freie Universität Berlin approved the study (approval number: 018.2021) following the latest version of the Declaration of Helsinki.

**TABLE 1:** Participants age and sex ratio per analysis.

	*N*	Female:male	Age
Behavioral analysis	61	28:33	22.44 (2.83)
Univariate task‐related cluster (localizer task)	67	34:33	22.49 (2.87)
Connectivity single task	64	32:32	22.61 (2.86)
Connectivity dual task	47	20:27	22.23 (2.80)
Connectivity single vs. dual task	45	18:27	22.27 (2.81)

*Note:* Showing means (standard deviation) for age and number of participants.

### 2.3 Experimental Procedure

Participants completed one online session and two fMRI sessions in the context of a practice intervention design. Here, we will focus on the results of fMRI session one (Figure 1A). Due to high drop‐out rates for the second (post‐practice) session and the strict head movement criteria for connectivity analyses (see below), the sample size was not large enough to analyze the practice‐related changes between the two sessions with sufficient power as it was initially planned in the pre‐registration (39 participants left, with 10, 13, and 16 per intervention group for the dual task).

The online session consisted of behavioral and cognitive measures and is part of a different study with a separate pre‐registration (https://osf.io/nfpqv). The two fMRI sessions (each 2.5–3 h) took place at the Cognitive Center for Neuroscience Berlin (CCNB). During the first session, participants started outside the scanner with a short familiarization of the modality‐pairing tasks (256 trials, 32 per single task, 64 per dual task). The following in‐scanner part started with a 10‐min resting‐state measurement with open eyes. It was followed by two runs of a localizer task, each containing single and dual tasks in both modality pairings, organized in a block design. Each run of the localizer task comprised six blocks, each containing 16 trials. The following two runs included only dual‐task trials. Each run consisted of 128 trials and was assigned to one modality pairing (i.e., modality‐compatible or modality‐incompatible). The remaining eight runs contained only single‐task trials in both modality pairings, with an easy and a difficult version of the task. The manipulation of task difficulty served as a control analysis for the MVPA and is not distinguished here. In each run, every task, modality pairing, and difficulty combination occurred only once, resulting in eight blocks with 16 trials per block. A visual overview of the run and task structure is presented in the Supporting Information (Figure S1). Details about the second session can be found in the pre‐registration of the study (https://osf.io/whpz8) and in Mueckstein et al. (2025).

### 2.4 Behavioral Tasks

Participants completed sensorimotor choice reaction tasks as single and dual tasks with two different modality pairings (modality‐compatible and modality‐incompatible, compare Figure 1B). The visual stimuli were a white square (pixel size 56.8 × 56.8) on a black background next to a fixation cross (pixel size 41.1 × 41.1, thickness 9.9 pixels) at six different positions (top, center, bottom, either right or left from the fixation cross). The auditory stimuli were pure tones at different frequencies (200, 450, and 900 Hz), presented in either the right or left ear. In the single‐task blocks, only one stimulus per trial was presented, while in the dual‐task blocks, one visual and one auditory stimulus were presented simultaneously (stimulus‐onset‐asynchrony = 0 ms). All stimuli were presented for 200 ms, followed by a response interval of 1500 ms and an inter‐stimulus interval of 200 ms. Each run concluded with an 8 s fixation period.

Participants were asked to indicate the side on which the stimulus was presented by either pressing a button with their right or left hand (index finger) and/or by saying the German word for “right” or “left.” The pairing of visual‐manual and auditory‐vocal is regarded as modality‐compatible, and the pairing of visual‐vocal and auditory‐manual as modality‐incompatible. Consequently, within each dual‐task condition, none of the pairings had a direct overlap between the presented stimulus modality and the required response modality. Nevertheless, they overlap between stimulus modality and the modality of the anticipated response‐related sensory consequences in the modality‐incompatible condition.

During the single‐task runs, we manipulated the task difficulty by adding visual noise, increasing the distance of the stimulus from the center, and reducing the contrast between the stimulus and background. Similarly, we added white noise for the auditory stimulus and reduced the volume. As preregistered, only easy blocks were used for the behavioral analysis to better compare the results to earlier studies. We did not distinguish between easy and difficult single‐task blocks for the fMRI analysis.

The order of the dual‐task runs was counterbalanced across participants, while the block position during each single‐task run was randomized for each participant. Each stimulus was presented equally often and in random order per trial within each block.

Data exclusion was determined per run. Specifically, participants were excluded if one of the following criteria applied to either one dual‐task run, more than one localizer run, or more than three single‐task runs: On a behavioral level, data were excluded if participants used the wrong response modality in a block for more than five trials (e.g., vocal response in VM block) or committed more than 30% errors (including omissions) in the single‐task or localizer runs. Due to the high error rate in the dual‐task runs (whole sample [*N* = 71] modality‐compatible, *M* = 19.68% [SD = 16.11, max = 73.44%], modality‐incompatible *M* = 42.48% [SD = 17.28, max = 73.44%]) we deviated from the pre‐registered protocol and applied the 30% criteria only to trials in which both stimuli required the same response location (i.e., congruent trials, averaged for both modality pairings in a dual‐task trial). This ensured that participants generally understood the task.

Further, we decided to deviate from the pre‐registration in the analysis of behavior. Specifically, we used a balanced integrated score (BIS, Liesefeld and Janczyk 2019; Mueckstein et al. 2025), which is defined as the difference between the z‐standardized reaction times and accuracies instead of separately analyzing reaction times and error rates. Liesefeld and Janczyk (2019) showed that the BIS parameter controls well for speed‐accuracy trade‐offs, thus accounting for different individual response strategies (i.e., focusing either more on accuracy or speed). Additionally, it reduces the number of analyses, which increases the statistical power and clarity of analyses. We used a standard paired *t*‐test together with a Bayesian paired *t*‐test to analyze the behavioral data.

Statistical analysis and plotting were conducted in R (version 4.2.2, R. C. Team 2020) with RStudio (version 2023.12.1, Rs. Team 2019), the tidyverse package (version 2.0.0, Wickham et al. 2019), and the BayesFactor package (version 0.9.12, Morey and Rouder 2024). Brain‐related plots were created with the ggseg package (version 1.6.5, Mowinckel and Vidal‐Piñeiro 2019), the brainconn package (version 0.1, Chopra et al. 2023), and one function from the CONN Toolbox (version 22a, Nieto‐Castanon and Whitfield‐Gabrieli 2022). The manuscript was created with the papaja package (version 0.1.2, Aust and Barth 2023).

### 2.5 MRI Data Acquisition and Preprocessing

#### 2.5.1 Acquisition

Due to a scanner upgrade at the imaging center, the data were acquired with two different MRI scanners. The first 25 participants were measured with Siemens Magnetom TIM TRIO syngo 3 T and the remaining participants with Siemens Magnetom 3.0 T Prisma, both with a 32‐channel head coil and based on the same parameters. At the end of the session, a high‐resolution T1‐weighted structural image was measured with 176 interleaved slices, 1 mm isotropic voxels; TE = 2.52 ms, TR = 1900 ms, FoV = 256 × 256 × 176 mm. The functional runs consisted of 139 whole‐brain echo‐planar images of 37 interleaved slices for the localizer task, and the dual‐task runs, and 183 whole‐brain echo‐planar images for each single‐task run. Each functional run was acquired with 3 mm isotropic voxels, TE = 30 ms, TR = 2000 ms, flip angle = 75°, FoV = 192 × 192 × 133 mm. After each dual‐task run, a gray‐field mapping was measured (3 mm isotropic voxel, TE1 = 4.92 ms and TE2 = 7.38 ms; TR = 400 ms; FoV = 192 × 192 × 133 mm, flip angle = 60°). Participants received auditory stimuli via MRI‐compatible headphones (Sensi‐Metrics S14, SensiMetrics, USA). Visual stimuli were projected on a screen at the end of the bore, which participants could view through a mirror attached to the head coil. Vocal responses were recorded via an MRI‐compatible microphone (OptimicTM MEG, Optoacoustics, Israel) and manual responses via MRI‐compatible 4‐button bimanual boxes (HHSC‐2x2, Current Designs, USA).

#### 2.5.2 Pre‐Processing

We converted the fMRI DICOM data into BIDS format, using dcm2bids (version 2.1.6, Boré et al. 2023) and applied the preprocessing pipeline of fMRIprep (version 21.0.2, Esteban et al. 2019), comprising 3D motion correction and slice‐time correction. Preprocessing contained the alignment of all functional data to a generated reference image, co‐registration, and the transformation to standard space. Anatomical T1 images were transformed into standard MNI space. The following boilerplate was created by fMRIprep:

Results included in this manuscript come from preprocessing performed using *fMRIPrep* 21.0.1 (Esteban et al. 2019; Esteban et al. 2020; RRID:SCR_016216), which is based on *Nipype* 1.6.1 (Gorgolewski et al. 2011; Gorgolewski et al. 2017; RRID:SCR_002502).

Preprocessing of B0 inhomogeneity mappings: A total of 2 fieldmaps were found available within the input BIDS structure for this particular subject. A *B0* nonuniformity map (or *fieldmap*) was estimated from the phase‐drift map(s) measured with two consecutive GRE (gradient‐recalled echo) acquisitions. The corresponding phase‐map(s) were phase‐unwrapped with prelude (FSL 6.0.5.1:57b01774).

Anatomical data preprocessing: A total of 1 T1‐weighted (T1w) images were found within the input BIDS dataset. The T1‐weighted (T1w) image was corrected for intensity non‐uniformity (INU) with N4BiasFieldCorrection (Tustison et al. 2010), distributed with ANTs 2.3.3 (Avants et al. 2008, RRID:SCR_004757), and used as T1w‐reference throughout the workflow. The T1w‐reference was then skull‐stripped with a *Nipype* implementation of the antsBrainExtraction.sh workflow (from ANTs), using OASIS30ANTs as the target template. Brain tissue segmentation of cerebrospinal fluid (CSF), white matter (WM), and gray matter (GM) was performed on the brain‐extracted T1w using fast (FSL 6.0.5.1:57b01774, RRID:SCR_002823, Zhang et al. 2001). Brain surfaces were reconstructed using recon‐all (FreeSurfer 6.0.1, RRID:SCR_001847, Dale et al. 1999), and the brain mask estimated previously was refined with a custom variation of the method to reconcile ANTs‐derived and FreeSurfer‐derived segmentations of the cortical gray matter of Mindboggle (RRID:SCR_002438, Klein et al. 2017). Volume‐based spatial normalization to one standard space (MNI152NLin2009cAsym) was performed through nonlinear registration with antsRegistration (ANTs 2.3.3), using brain‐extracted versions of both T1w reference and the T1w template. The following template was selected for spatial normalization: *ICBM 152 Nonlinear Asymmetrical template version 2009c* (Fonov et al. 2009, RRID:SCR_008796; TemplateFlow ID: MNI152NLin2009cAsym).

Functional data preprocessing: For each of the 23 BOLD runs found per subject (across all tasks and sessions), the following preprocessing was performed. First, a reference volume and its skull‐stripped version were generated using a custom methodology of *fMRIPrep*. Head‐motion parameters with respect to the BOLD reference (transformation matrices and six corresponding rotation and translation parameters) are estimated before any spatiotemporal filtering using mcflirt (FSL 6.0.5.1:57b01774, Jenkinson et al. 2002). BOLD runs were slice‐time corrected to 0.978 s (0.5 of slice acquisition range 0–1.96 s) using 3dTshift from AFNI (Cox 1996, RRID:SCR_005927). The BOLD time‐series (including slice‐timing correction when applied) were resampled onto their original, native space by applying the transforms to correct for head‐motion. These resampled BOLD time‐series will be referred to as *preprocessed BOLD in original space*, or just *preprocessed BOLD*. The BOLD reference was then co‐registered to the T1w reference using bbregister (FreeSurfer), which implements boundary‐based registration (Greve and Fischl 2009). Co‐registration was configured with six degrees of freedom. Several confounding time‐series were calculated based on the *preprocessed BOLD*: framewise displacement (FD), DVARS, and three region‐wise global signals. FD was computed using two formulations following Power (absolute sum of relative motions, Power et al. (2014)) and Jenkinson (relative root mean square displacement between affines, Jenkinson et al. 2002). FD and DVARS are calculated for each functional run, both using their implementations in *Nipype* (following the definitions by Power et al. 2014). The three global signals are extracted within the CSF, the WM, and the whole‐brain masks. Additionally, a set of physiological regressors were extracted to allow for component‐based noise correction (*CompCor*, Behzadi et al. 2007). Principal components are estimated after high‐pass filtering the *preprocessed BOLD* time‐series (using a discrete cosine filter with 128 s cut‐off) for the two *CompCor* variants: temporal (tCompCor) and anatomical (aCompCor). tCompCor components are then calculated from the top 2% variable voxels within the brain mask. For aCompCor, three probabilistic masks (CSF, WM, and combined CSF + WM) are generated in anatomical space. The implementation differs from that of Behzadi et al. in that instead of eroding the masks by 2 pixels on BOLD space, the aCompCor masks are subtracted by a mask of pixels that likely contain a volume fraction of GM. This mask is obtained by dilating a GM mask extracted from FreeSurfer's *aseg* segmentation, and it ensures components are not extracted from voxels containing a minimal fraction of GM. Finally, these masks are resampled into BOLD space and binarized by thresholding at 0.99 (as in the original implementation). Components are also calculated separately within the WM and CSF masks. For each CompCor decomposition, the *k* components with the largest singular values are retained, such that the retained components' time series are sufficient to explain 50% of variance across the nuisance mask (CSF, WM, combined, or temporal). The remaining components are dropped from consideration. The head‐motion estimates calculated in the correction step were also placed within the corresponding confounds file. The confound time series derived from head motion estimates and global signals were expanded with the inclusion of temporal derivatives and quadratic terms for each (Satterthwaite et al. 2013). Frames that exceeded a threshold of 0.5 mm FD or 1.5 standardized DVARS were annotated as motion outliers. The BOLD time‐series were resampled into standard space, generating a *preprocessed BOLD run in MNI152NLin2009cAsym space*. First, a reference volume and its skull‐stripped version were generated using a custom methodology of *fMRIPrep*. All resamplings can be performed with *a single interpolation step* by composing all the pertinent transformations (i.e., head‐motion transform matrices, susceptibility distortion correction when available, and co‐registrations to anatomical and output spaces). Gridded (volumetric) resamplings were performed using antsApplyTransforms (ANTs), configured with Lanczos interpolation to minimize the smoothing effects of other kernels. Non‐gridded (surface) resamplings were performed using mri_vol2surf (FreeSurfer). Many internal operations of *fMRIPrep* use *Nilearn* 0.8.1 (Abraham et al. 2014, RRID:SCR_001362), mostly within the functional processing workflow. For more details of the pipeline, see (the section corresponding to workflows in *fMRIPrep*'s documentation) (https://fmriprep.readthedocs.io/en/latest/workflows.html “FMRIPrep's documentation”).

### 2.6 Copyright Waiver

The above boilerplate text was automatically generated by fMRIPrep with the express intention that users should copy and paste this text into their manuscripts *unchanged*. It is released under the [CC0] (https://creativecommons.org/publicdomain/zero/1.0/) license.

### 2.7 fMRI Data Analysis

#### 2.7.1 Univariate Region‐of‐Interest Definition

We conducted the first‐level analysis in SPM12 on the two normalized and smoothed (8 mm FWHM Gaussian) BOLD runs of the localizer tasks with a block design to define activity‐based task‐related clusters. We included six motion parameters (3× rotation, 3× translation) and the framewise displacement parameter as regressors of no interest in the general linear model. We calculated the statistical parametric maps for each participant, including contrasting the stimulus modalities (visual vs. auditory), the response modalities (manual vs. vocal), and the task type (single vs. dual task). On the second level, individual brain maps were averaged and tested voxel‐wise using a one‐sample *t*‐test between the defined contrasts. We used a cluster‐wise FWE‐corrected significant threshold of p=0.05 on the voxel level. For the single versus dual‐task contrast, we restricted the cluster selection to the frontal lobe, as previous studies on dual‐tasking consistently showed frontal activity using the same contrast. This process resulted in one task‐based activity cluster per hemisphere for visual, auditory, manual, vocal, and dual tasks (frontal). In case there was more than one remaining cluster, we selected the one with the maximum intensity.

#### 2.7.2 Preprocessing Functional Brain Network Connectivity

We followed the preprocessing steps described by Thiele et al. (2022), and in that, the recommendations of Parkes et al. (2018) (pipeline No. 6). The final bold time series were created by using the “app‐fmri‐2‐mat” code locally, provided by Josh Faskowitz (https://github.com/faskowit/app‐fmri‐2‐mat). In this code, the nuisance regression strategy using the parameters calculated by the fMRIprep confound file was applied. For the following parameters, the raw values along with all three derivatives were used: global signal, cerebrospinal fluid, white matter, 3× translation, 3× rotation, and framewise displacement. In addition, mean task‐evoked activation was removed using the basis‐set task regressors as suggested by Cole et al. (2019) to correct for an inflation of the correlation between brain regions. This step removes the activity evoked by the timing of task events in each region individually without affecting the measure of interest, which is the potential interaction between two regions in response to task demands (compare fig. 1 in Cole et al. 2019). A task run was only included in the analysis if the mean framewise displacement was below 0.2 mm, the proportion of spikes larger than 0.25 mm was below 20%, and if there were no spikes above 5 mm (Parkes et al. 2018; Thiele et al. 2022). In addition, the behavioral performance criteria described above (“Behavioral tasks”) must also be fulfilled.

Deviating from the pre‐registration, we applied the schaefer200 instead of the schaefer100 parcellation (Schaefer et al. 2018) for more fine‐grained insights. Note that both parcellations showed the same results for the two pre‐registered analyses and widely overlapping results for the unregistered analysis. The results with the pre‐registered parcellation schaefer100 can be found in the Supporting Information (Tables S4 and S5). All schaefer regions were assigned to one out of seven functional brain networks (Yeo et al. 2011): visual, somatomotor, dorsal attention, saliency‐ventral attention, limbic, control, default.

**FIGURE 2:**
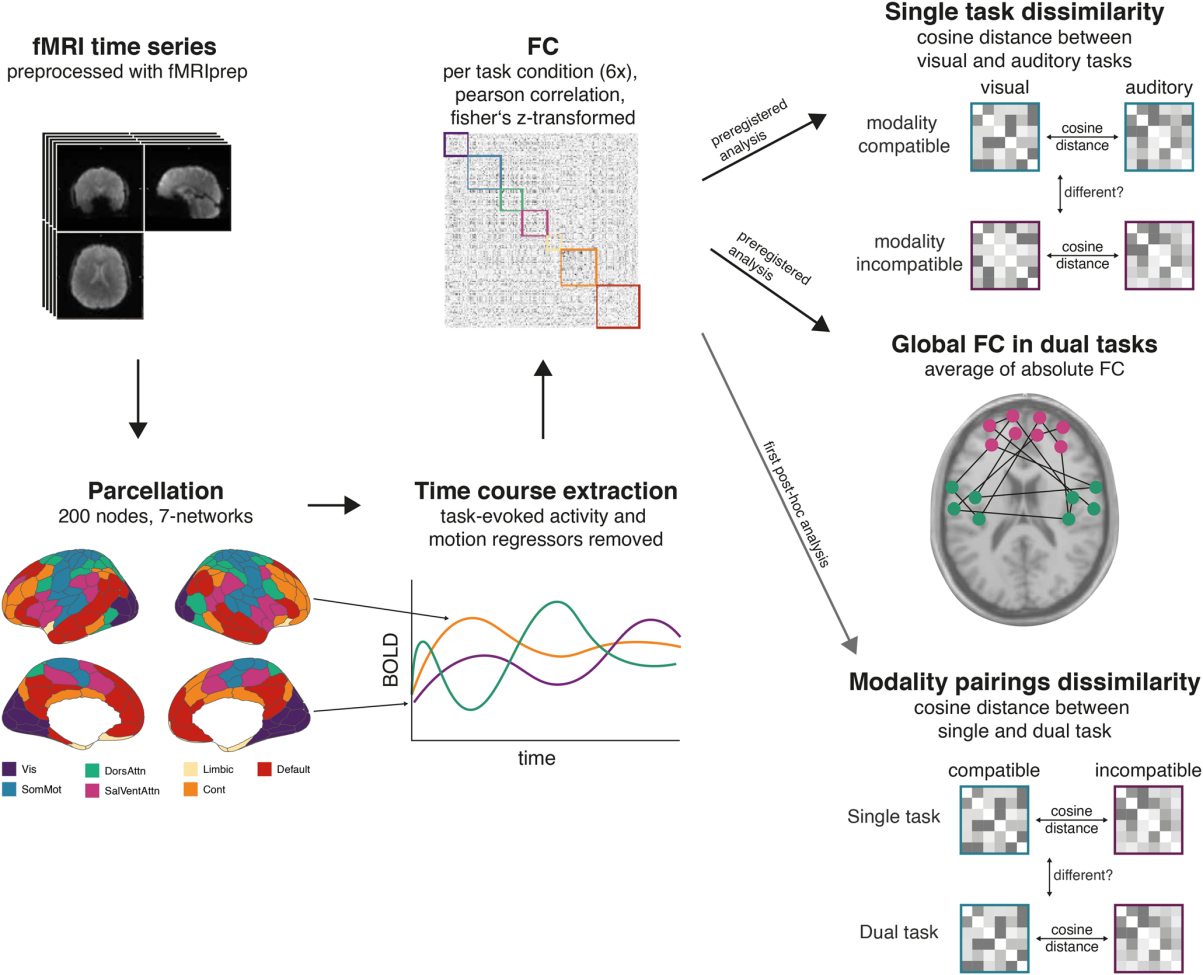
Processing pipeline from preprocessed data to functional connectivity matrix with the two preregistered analyses and one of two unregistered post hoc analysis.

The resulting time series per run and participant for the single tasks were then filtered for each single task and averaged over runs to account for potential differences between runs. Functional connectivity matrices (FC) were computed as Fisher z‐transformed Pearson correlations between all 200 regions. Accordingly, the Pearson correlation defines the strength of the connection between two regions. This procedure resulted in six different FC matrices: visual‐manual, auditory‐vocal, visual‐vocal, auditory‐manual, modality‐compatible dual task, and modality‐incompatible dual task. Figure 2 provides an overview of the preprocessing steps.

#### 2.7.3 Dissimilarity of Whole‐Brain Functional Connectivity

To investigate the first research question, whether whole‐brain single‐task connectivity patterns differ between modality pairings, we employed the cosine distance as a measure of the dissimilarity between FCs. This measure represents the angle (cosine) between two vectors without considering a potential difference in length (Han et al. 2012):

$$d_{\cos}\left(a,b\right)=1-\frac{\sum_{n}^{i=1}a_{i}b_{i}}{\left\|a\|\|b\right\|},$$

where *a* and *b* are connection vectors and *n* is the total number of connections (compare Thiele et al. 2022).

We compared the brain FC dissimilarity between the two modality‐compatible single tasks (visual‐manual and auditory‐vocal) with the brain FC dissimilarity of the two modality‐incompatible single tasks (visual‐vocal and auditory‐manual). Please note that due to the simultaneous presentation of the two tasks in dual‐task blocks, we are not able to investigate such an overlap between component tasks in dual‐task blocks. Participants were excluded from this analysis if they showed extensive head movement in more than six (out of eight) single‐task runs.

#### 2.7.4 Global Functional Connectivity Analysis

Our second research question targeted the dual‐task FC, with the hypothesis that the modality‐incompatible dual task requires more fronto‐parietal control‐related interactions compared to the modality‐compatible dual task, which could reflect the higher dual‐task costs in behavioral outcomes. In previous research, authors could not find any differences in performance between the corresponding single tasks, only between dual tasks (Hazeltine et al. 2006; Schacherer and Hazeltine 2020; Stelzel et al. 2006). Accordingly, we focused this analysis only on the dual‐task blocks where modality‐based crosstalk actually emerges. The dissimilarity analysis used for the single task can not answer this question about a difference in FC strength, as the cosine distance is a measure of overlap and does not reveal differences in strength. Thus, for each of the dual‐task matrices, we calculated the whole‐brain FC as the average of the absolute connections within and between all network combinations, while we averaged the between connections for each network (resulting in seven within and seven between network combinations). For each of the network combinations, we applied a linear mixed‐effects model, fitted with log‐likelihood maximization, with participant and modality pairing as random effects (nlme package, function *lme*, version 3.1‐160, Pinheiro et al. 2022). This allows us to assess the difference between modality pairings, while controlling for the factors age, gender, and head movement. Additionally, we calculated the Bayesian Factor, using a paired *t*‐test between the two modality pairings, for the seven within and the 21 between‐network combinations.

#### 2.7.5 Unregistered Post Hoc Functional Similarity Analysis

As unregistered post hoc (not pre‐registered) analysis, we averaged the single‐task matrices for each modality pairing to further compare the FC dissimilarity between dual‐task modality pairings with the FC dissimilarity of the single‐task modality pairings (see Figure 2).

For both pre‐registered FC dissimilarity analysis and unregistered post hoc, we calculated the FC dissimilarity for each within and between functional network pairing. The between‐network combinations were averaged per network, resulting in seven within‐network and seven between‐network combinations. The same model and additional analysis as for the dual‐task matrices were calculated for the FC dissimilarity by controlling for the number of valid runs in the single tasks.

#### 2.7.6 Unregistered Post Hoc Raw Functional Connectivity Analysis and Correlation

**FIGURE 3:**
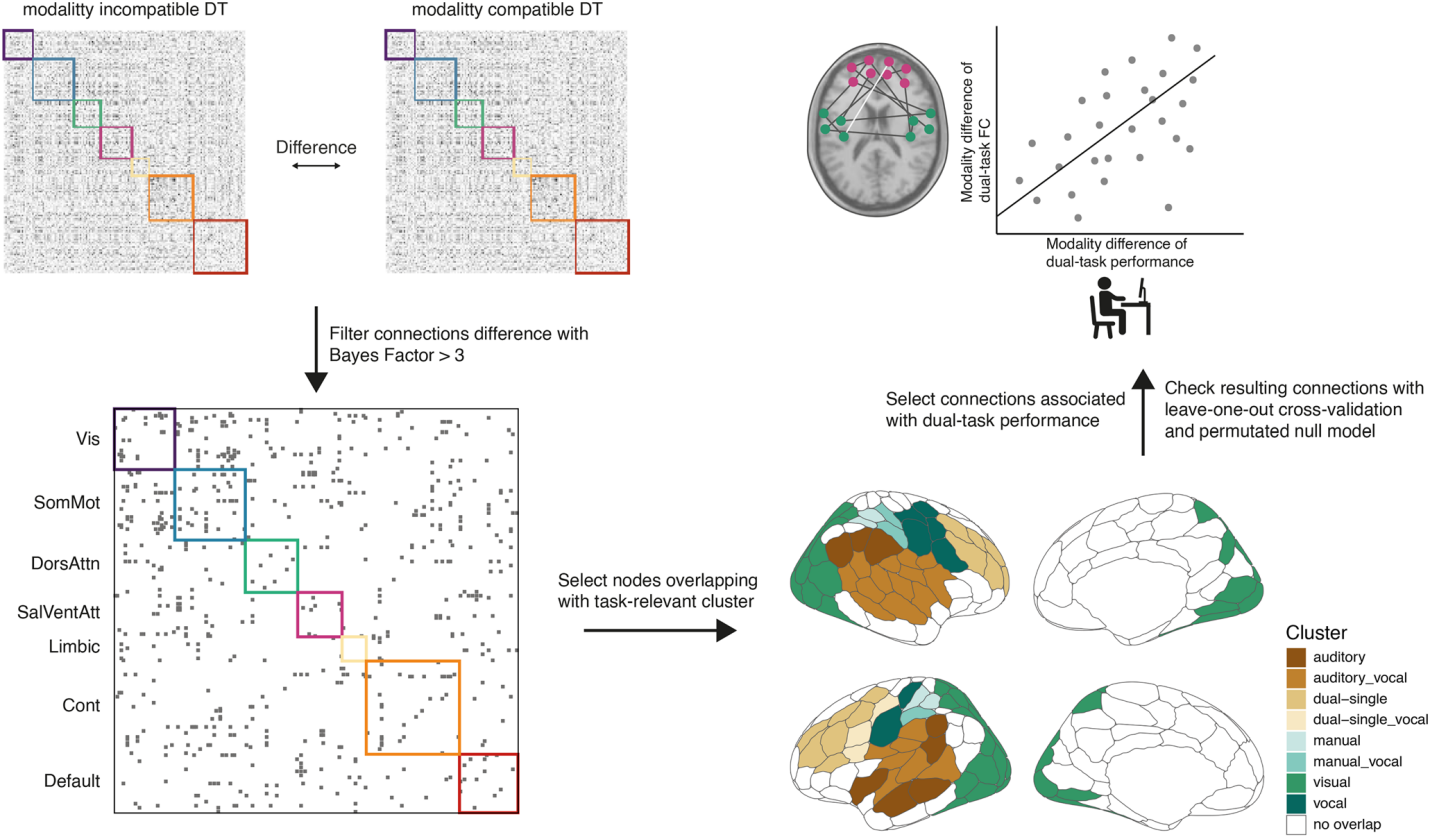
Processing steps for the second unregistered post hoc analysis. We first calculated the difference between modality pairings of the dual‐task FC matrices, next the difference was tested against zero and only connections with Bayes Factor above 3, favoring a difference remained. To relate the regions to our task‐specific activity clusters we only selected regions that overlapped. Finally, we focus only on connections with a significant correlation (p<0.05) between the modality difference between dual‐task performance and FC difference. The selection of connections based on the correlation was confirmed by a leave‐one‐out cross‐validation approach and compared to a permutated null distribution of rho‐values.

As additional unregistered post hoc analyses, we calculated the difference between the FC matrix of the modality‐incompatible dual task and the modality‐compatible dual task per participant. The resulting 200 × 200 difference matrix was tested against zero, applying a Bayesian one‐sample *t*‐test (*ttestBF* function in the r‐package BayesFactor) and filtered for connections with a Bayes Factor above 3, which indicates moderate evidence for the alternative hypothesis (Andraszewicz et al. 2015). See Figure 3 for a visual overview of these steps. As we were mainly interested in task‐specific differences, only connections between regions that overlapped with the univariate task‐relevant cluster from the localizer task were considered. The Supporting Information reports the whole‐brain FC matrix filtered only by the Bayes Factor (compare Figure S2).

Finally, to investigate which connection differences are related to the behavioral difference, we calculated partial Spearman correlation coefficients between behavioral performance (difference between modality pairings for the BIS parameter) and the FC difference between modality pairings (all connections). We controlled for differences in age, gender, and framewise displacement.

Note that for the final interpretation and discussion, we focus on the connections significantly correlated with behavior (uncorrected p<0.05) and report the parameter rho for each connection.

We assessed the robustness of these selective connections by applying a leave‐one‐out cross‐validation procedure. We repeated our analysis steps *N* times (sample size was N=47), removing each time the data from one participant. On each iteration, we only selected connections different from zero (${\mathrm{BF}}_{10}>3$) and brain regions overlapping with task‐related clusters. Those remaining connections were correlated with the behavioral difference scores. For each iteration, we compared the resulting *rho*‐values with the distribution of *rho*‐values under the null model. This null model distribution was created by randomly removing one participant (as the leave‐one‐out cross‐fold was also based on *N* − 1 participants) and permutating the behavioral vector before calculating the Spearman correlation between FC difference and behavioral difference score. We repeated this procedure 10,000 times for each connection. We further compared the resulting 47 *rho*‐values from the cross‐validation with the null distribution and selected only connections for which all the 47 *rho*‐values fell below the 5% percentile, or above the 95% percentile.

## 3 Results

### 3.1 Pre‐Registered Analysis

#### 3.1.1 Robust Difference Between Modality Pairings in Behavioral Dual‐Task Costs

We combined reaction times and accuracies into the balanced integration score (BIS) (Liesefeld and Janczyk 2019; Mueckstein et al. 2025) to account for the dependency between the two behavioral performance parameters (see averaged reaction times and accuracies per task type and modality pairing in Table 2). Please note that the behavioral data were already reported in Mueckstein et al. (2025), focusing on group‐specific practice effects using a subsample of the current sample. Dual‐task costs were calculated as the difference between single and dual tasks, where higher BIS scores correspond to higher dual‐task costs (i.e., higher reaction times and error rates in dual tasks compared to single tasks). The modality pairings differed significantly, *t*(60) = −14.36, *p* < 0.001 with higher dual‐task costs for the modality‐incompatible pairing (*M* = 4.13, SE = 0.14) compared to the modality‐compatible pairing (*M* = 2.03, SE = 0.14). The Bayes factor confirmed the strong difference as providing extreme evidence, M=−2.08, 95% HDI $\left[-2.38,-1.79\right]$, ${\mathrm{BF}}_{10}=1.05\times 10^{18}$ (see Figure 1C). This result replicates several previous findings in the field, demonstrating that performance with modality‐incompatible pairings in the dual task is more error‐prone and slower compared to performance with modality‐compatible pairings (Göthe et al. 2016; Hazeltine et al. 2006; Mueckstein et al. 2022; Stelzel et al. 2006).

**TABLE 2:** Reaction times and error rates per task type, and modality mapping.

	Mean	SD	SE
Reaction times (ms)			
Dual task			
Modality‐compatible	751.92	193.98	2.45
Modality‐incompatible	868.59	229.92	3.41
Single task			
Modality‐compatible	508.07	241.14	1.99
Modality‐incompatible	470.59	170.65	1.41
Error rate (%)			
Dual task			
Modality‐compatible	17.23	14.35	1.32
Modality‐incompatible	34.31	18.94	1.74
Single task			
Modality‐compatible	2.75	6.30	0.21
Modality‐incompatible	2.47	6.01	0.20

*Note:* Showing means, standard deviation, and standard error for reaction times and error rates. Single tasks were averaged across the corresponding component tasks (VM + AV for modality‐compatible and VV + AM for modality‐incompatible).

#### 3.1.2 No Difference in Single‐Tasks FC Dissimilarity Between Modality Pairings

**FIGURE 4:**
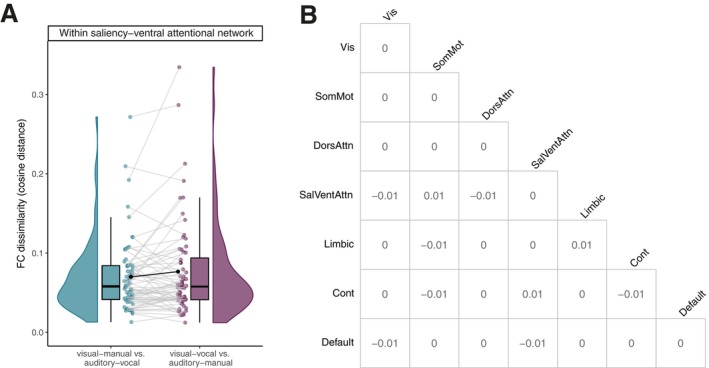
FC dissimilarity between single‐task modality pairings. (A) FC dissimilarity within the ventral attentional network per modality pairing during single task. The graph depicts distribution, boxplot, individual data, and the mean for each modality pairing of the cosine distance (not corrected for age, gender, and framewise displacement). We detected no significant difference in FC dissimilarity (cosine distance) between the two modality pairings. (B) Matrix shows the difference between modality pairings for the FC dissimilarity for each network combination. The diagonal of the matrix describes the within‐network difference. We identified no significant difference for any of the network combinations. Cont = control network, Default = default mode network, DorsAttn = dorsal attentional network, Limbic = limbic network, SalVentAttn = saliency‐ventral attentional network, SomMot = somato‐motor network, Vis = visual network.

To address our first neural research question of whether the single‐task FC pattern overlaps more strongly for modality‐incompatible pairings than for modality‐compatible pairings, we compared the network FC dissimilarity between modality pairings in single tasks. There was no significant effect of modality pairings in single‐task FC dissimilarity in any network combination (see Figure 4B). The highest *t*‐value for the factor modality pairing was observed for the FC dissimilarity within the ventral attentional network (*p*‐value uncorrected), $t\left(63\right)=1.53$, p=0.132. This is confirmed by a paired Bayesian *t*‐test, which provided anecdotal to moderate evidence for the hypothesis that there is no difference between the two modality pairings (compare Table 3 for all network combinations). Figure 4A depicts the difference between modality pairings within the ventral attentional network. Thus, we observed no evidence that the single‐task FC for modality‐incompatible tasks (i.e., AM, VV) overlap differently than those for modality‐compatible tasks (i.e., VM, AV).

**TABLE 3:** Bayes paired *t*‐test between FC dissimilarity (cosine distance) of the single‐task modality pairings.

	Network	Bayes factor	Error
Between network	control	0.137	0.000915
	default	0.148	0.000873
	dorsAtt	0.156	0.000843
	limbic	0.194	0.000729
	somatomotor	0.137	0.000915
	ventrAtt	0.139	0.000909
	visual	0.287	0.000561
Within network	control	0.420	0.000430
	default	0.291	0.000556
	dorsAtt	0.139	0.000908
	limbic	0.187	0.000750
	somatomotor	0.239	0.000634
	ventrAtt	0.426	0.000426
	visual	0.166	0.000810

*Note:* Task‐activity regressed out, schaefer200 parcellation.

#### 3.1.3 No Difference in FC Strength Between Dual Tasks

**FIGURE 5:**
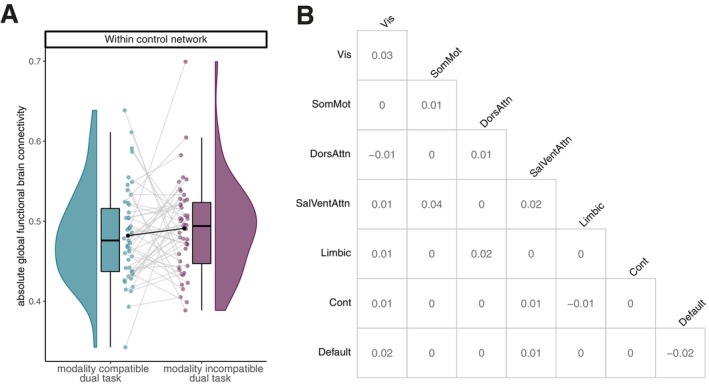
Whole‐brain functional connectivity between dual‐task modality pairings. (A) Absolute whole‐brain functional connectivity within the control network per modality pairing during dual task. The graph shows the distribution, boxplot, individual data, and the mean for each modality pairing of the averaged absolute FC (not corrected for age, gender, framewise displacement). We found no significant difference between the dual‐task modality pairings. (B) Difference between modality pairings for the absolute whole‐brain functional connectivity for each network combination. The diagonal of the matrix describes the within‐network difference. We found no significant difference for any of the network combinations.

In our second research question, we further examined the involvement of the control network during dual‐task performance by comparing the absolute averaged functional connectivity for each within‐ and between‐network combination between the modality pairings. With this analysis, we investigate whether the connectivity strength of fronto‐parietal regions associated with cognitive control processes in dual tasks (Worringer et al. 2019) differ between the modality‐compatible and the modality‐incompatible pairing. There was no significant effect of modality pairings in functional connectivity during dual‐task performance in any brain network combination (compare Figure 5). The highest *t*‐value for the factor modality pairing was observed for functional connectivity within the ventral attentional network (*p*‐value uncorrected), $t\left(46\right)=1.46$, p=0.150. In line with this, Bayes factors provided anecdotal to moderate evidence for the hypothesis that there is no difference between the two modality pairings during dual‐task (compare Table 4 for all Bayes Factor per network combination). These results do not confirm the assumption of a higher connectivity strength of the control network or any other network during performance of the modality‐incompatible dual task compared to the modality‐compatible dual task. This null result is surprising, considering the consistent involvement of frontal regions in dual‐task situations and the robust behavioral modality‐compatibility effect.

**TABLE 4:** Bayes paired *t*‐test between absolute functional connectivity of dual‐task modality pairings.

	Network	Bayes factor	Error
Between network	control	0.220	0.000546
	default	0.158	0.000638
	dorsAtt	0.163	0.000630
	limbic	0.162	0.000632
	somatomotor	0.201	0.000570
	ventrAtt	0.267	0.000495
	visual	0.252	0.000510
Within network	control	0.210	0.000559
	default	0.390	0.000404
	dorsAtt	0.165	0.000626
	limbic	0.159	0.000637
	somatomotor	0.173	0.000613
	ventrAtt	0.443	0.000377
	visual	0.278	0.000485

*Note:* Task‐activity regressed out, schaefer200 parcellation.

We repeated both analyses with multiple variations and graph measures to rule out that our null results are specific to our preprocessing and analysis decisions (Kristanto et al. 2024). Specifically, we analyzed the data by separately changing one of the following properties: schaefer100 instead of schaefer200 parcellation (compare Table S4), preprocessing the data without regressing out task‐related brain activity (Table S6), using Pearson correlation and Euclidean distance (compare Table S2) instead of cosine distance as FC dissimilarity measures and the relative average of the whole‐brain functional connectivity (compare Table S3), instead of the absolute. Also, we separately compared the brain graph's modularity (as a measure of network segregation, compare Figure S8 and Table S7) and global efficiency (as a measure of network communication, compare Figure S9 and Table S8) between modality pairings for single and dual tasks to directly compare the network reconfiguration. All analyses demonstrate the same results as presented above: no difference in single‐task overlap and no different involvement of the control network during dual tasks, which suggests that these global network attributes do not provide the basis for understanding the neural underpinnings of the robust behavioral modality‐compatibility effect.

However, considering the robust behavioral effect of modality pairings, we further explored the data with additional analyses. First, we investigated whether the FC dissimilarity analysis is able to detect potentially larger differences between modality pairings within each task type (i.e., comparing FC dissimilarity within single tasks and dual tasks). Based on the behavioral effects, we assume that the average single‐task connectivity patterns for each modality mapping are more similar to each other compared to the dual‐task connectivity pattern.

### 3.2 Unregistered Post Hoc Analyses

#### 3.2.1 Significant Difference Between Single and Dual Task FC Dissimilarity of Modality Pairings

Applying a similar approach as for the modality‐compatible versus modality‐incompatible single tasks, we compared the FC dissimilarity of modality pairings between single tasks and dual tasks. This allows us to investigate whether the FC dissimilarity between modality pairings is different between single and dual tasks, similar to the behavioral data, where we usually do not see a difference in single tasks but a robust difference in dual tasks. First, we averaged the single‐task FC matrix per modality pairing (resulting in one FC for modality‐compatible single tasks and one for modality‐incompatible single tasks) to obtain the same data structure as for the dual tasks. We then tested if the distance between the modality‐compatible and the modality‐incompatible FC differs between single tasks and dual tasks. We used the same model as for the single‐task FC dissimilarity analysis and compared again within and between networks.

**FIGURE 6:**
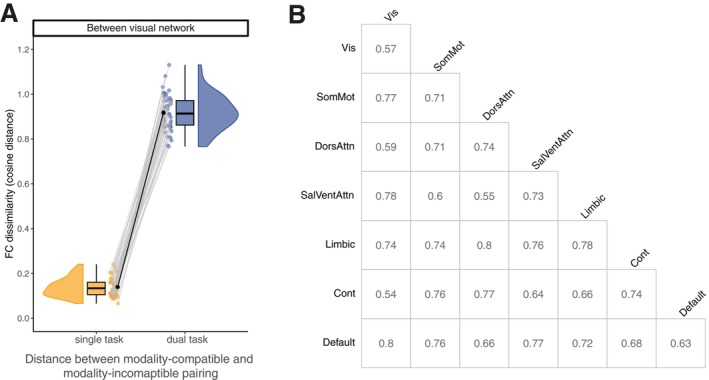
FC dissimilarity between modality pairings for single and dual tasks. (A) FC dissimilarity between modality pairing between the visual network and all other networks per task type. The graph provides distribution, boxplot, individual data, and the mean for each task type. We identified a significant difference in FC dissimilarity (cosine distance) of modality pairings between single and dual tasks. (B) Matrix demonstrates the difference between single and dual tasks for the cosine distance between modality‐compatible and modality‐incompatible for each network combination. The diagonal of the matrix describes the within‐network difference. We identified a significant difference for all of the network combinations.

The linear mixed‐effects model revealed significant differences between single‐task and dual‐task FC dissimilarity in all within and between network combinations (all p<0.001, BH corrected), with higher values for the dual task (averaged over the between‐network combinations for each network), *M* = 0.75, SE = 0.011, compared to the single task, *M* = 0.08, SE = 0.004. The smallest *t*‐value for the factor task type was observed for the FC dissimilarity within the dorsal attentional network, $t\left(43\right)=-30.15$, p<0.001. Compare Figure 6 for the differences between task types for all network combinations. Bayes factors confirmed this effect, providing strong evidence for the difference between single tasks and dual tasks (paired Bayes *t*‐test, all ${\mathrm{BF}}_{10}>3.96\times 10^{19}$, compare Table 5 for all network combinations). We repeated this analysis for the schaefer100 parcellation and could replicate the significant difference between single and dual‐task in all network combinations (compare Table S5 and Figure S3).

**TABLE 5:** Bayes paired *t*‐test between similarity (cosine distance) of single and dual tasks.

	Network	Bayes factor	Error
Between network	control	4.02e+38	4.36e−42
	default	2.91e+39	3.69e−43
	dorsAtt	2.94e+35	5.19e−39
	limbic	1.43e+38	7.29e−42
	somatomotor	6.34e+37	2.81e−41
	ventrAtt	2.19e+34	3.27e−38
	visual	8.37e+38	1.08e−42
Within network	control	3.63e+30	1.17e−35
	default	3.04e+29	1.70e−33
	dorsAtt	3.96e+19	8.80e−24
	limbic	5.82e+21	3.10e−27
	somatomotor	9.72e+24	7.86e−29
	ventrAtt	2.40e+20	1.76e−24
	visual	2.03e+22	1.23e−26

*Note:* Task‐activity regressed out, schaefer200 parcellation.

These results imply two aspects. First, FC dissimilarity of the modality pairings is significantly higher for dual compared to single tasks. Second, when comparing the absolute values of the cosine distance, we observed values close to 0 (vectors are proportional to each other, as $1-\cos\left(0^{{}^{\circ}}\right)=1-1=0$) for the two averaged single tasks. This indicates that the modality‐compatible single‐task FC is rather similar to the modality‐incompatible single‐task FC. In contrast, the dual‐task values were close to 1 (vectors are orthogonal to each other, as $1-\cos\left(90^{{}^{\circ}}\right)=1-0=1$), suggesting a substantial distance between the modality‐compatible dual task and the modality‐incompatible dual task. Since our findings of the preregistered analyses on the network level, as well as on the whole‐brain graph analysis, revealed no difference in connectivity strength, nor in network segregation or network communication between dual‐task pairings, we aimed to further understand this high FC dissimilarity between the two dual‐task FCs and, particularly, examined which characteristics of this difference are associated with behavioral differences. As we presume that our previous analysis on whole‐brain and network‐level might have been too global to detect subtle differences between modality pairings, we conducted an additional unregistered post hoc analysis considering the raw dual‐task FCs on a connectivity level.

#### 3.2.2 Characterizing the Difference Between Dual‐Task Functional Connectivity

**FIGURE 7:**
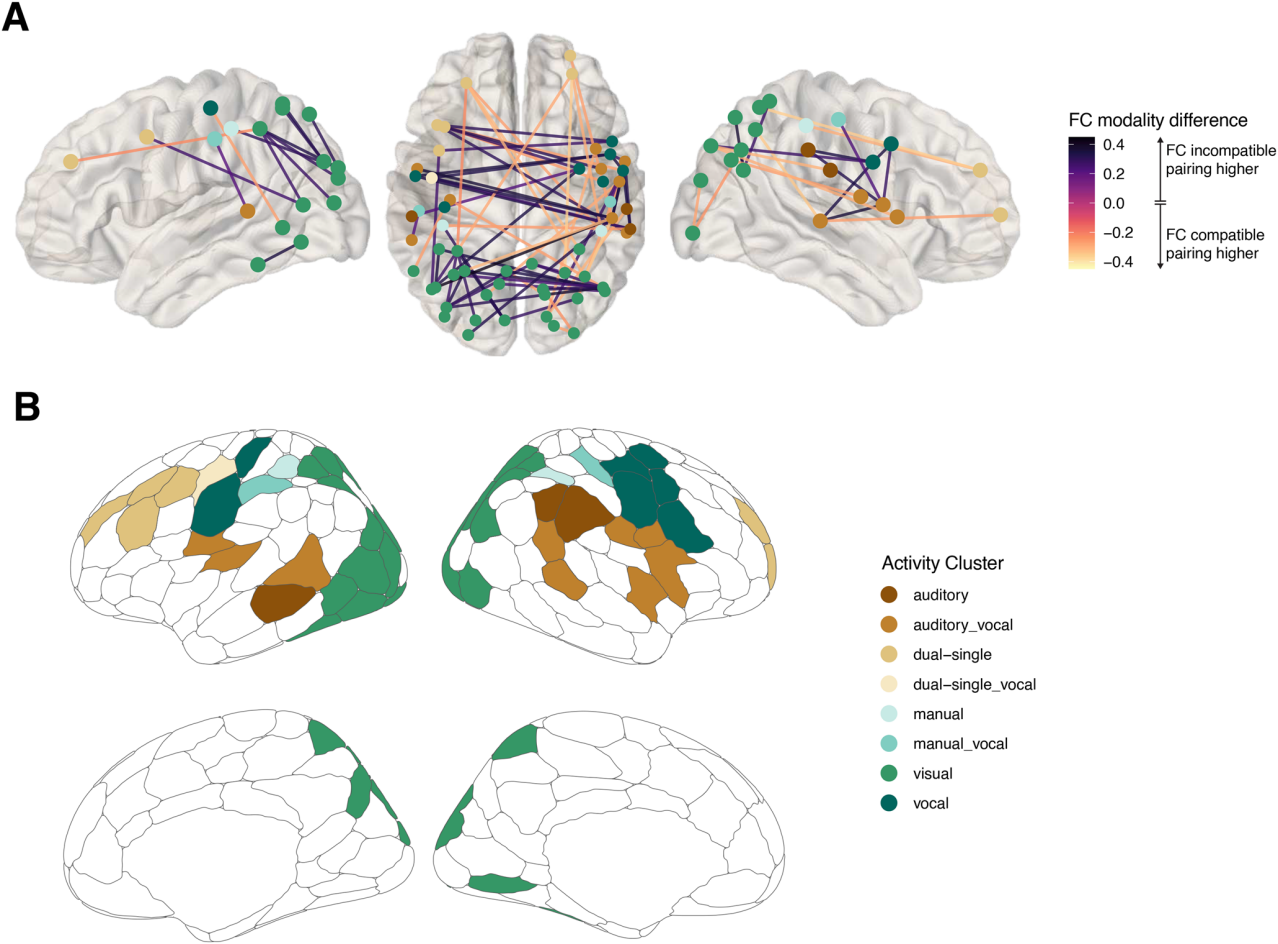
Significant different connections between modality pairings during dual tasks. (A) The graph depicts the connections, which are significantly different between the dual‐task modalities, restricted to regions overlapping with task‐related activity clusters. Color of regions indicate the corresponding activity cluster, color of connections the difference value of functional connectivity (FC) between modality‐incompatible dual task (positive values) and modality‐compatible dual task (negative values). The two sagital views contain only regions and connections within the corresponding hemisphere. The superior view contains all significant connections. (B) The graph depicts the schaefer200 parcellation in medial and sagital view. Color of the regions indicate the corresponding task‐related activity clusters with which the region overlaps. White regions did not overlap with any of our activity clusters.

**FIGURE 8:**
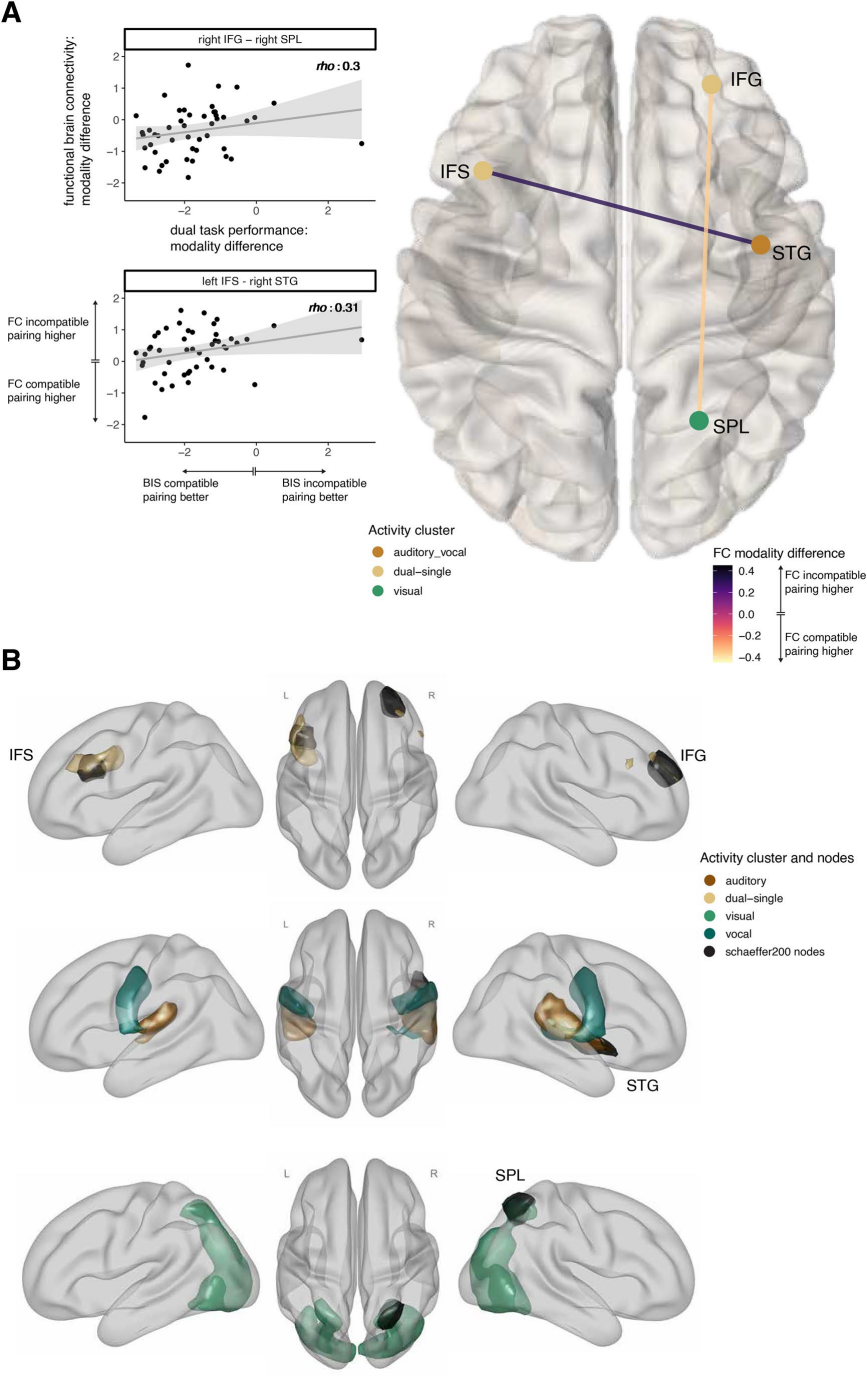
Relation between dual‐task behavior and functional connectivity. (A) The superior view of the brain with the two connections significantly different between dual‐task modalities, significantly related to the dual‐task behavior and validated by a leave‐one‐out cross‐validation. The color of regions indicates the corresponding activity cluster, and the color of connections indicates the difference value of functional connectivity (FC) between modality‐incompatible dual task (positive values) and modality‐compatible dual task (negative values). Each scatter plot corresponds to one connection and depicts each individual as one point, whereas the *y*‐axis represents the difference score between modalities of the functional connectivity during dual tasks and the *x*‐axis the difference score between modalities of the behavioral performance during dual task, operationalized as BIS parameter. The corresponding rho‐value is based on the partial correlation, corrected for age, gender, and framewise displacement. (B) Anatomical location of relevant schaefer200 regions for brain‐behavior relation, together with the task‐based activity clusters. Labels correspond to the Schaefer regions, colors to the activity clusters. Label abbreviations: IFG = inferior frontal gyrus, STG = superior temporal gyrus, SPL = superior parietal lobule, IFS = inferior frontal sulcus.

We calculated the difference between modality‐compatible and modality‐incompatible FC per participant and selected only the connections with a robust (${\mathrm{BF}}_{10}>3$) difference between pairings during dual‐task performance. Further, we focused on those brain regions from the schaefer200 parcellation, which overlap with the clusters a priori identified as task‐related (univariate analysis of the localizer task), as we were interested in the neural mechanisms of the modality‐compatibility effect and aimed at identifying which specific functional brain connections contributed to this effect. The remaining connections are depicted in Figure 7A and an overview of the location of each region in the task‐related clusters in Figure 7B. On a descriptive level, it is remarkable that after this selection process, more interhemispheric connections between frontal regions and temporoparietal regions remain, together with highly connected regions, which overlap with the visual cluster. Both showed significantly higher FC during the modality‐incompatible dual task compared to the modality‐compatible dual task (purple connections). On the other hand, we observed more anterior–posterior connections with a higher FC during the modality‐compatible dual task compared to the modality‐incompatible dual task. To identify connections relevant for behavior, we calculated partial Spearman correlations between FC differences and behavioral difference scores between both dual tasks, controlling for age, gender, and framewise displacement (for an overview of connections significantly related to performance without the requirement that they are significantly different between modality pairings, see Figure S2). Filtering the remaining connections for their relation to dual‐task performance (uncorrected p<0.05) revealed two connections (see Figure 8A). We decided to use the uncorrected *p*‐values for this selection, as we assumed very small correlation values as typical for brain‐behavior correlations (Marek et al. 2022) and wanted to be as sensitive as possible after the null results on a network level. The first connection was present between the left inferior frontal sulcus (IFS) and the right superior temporal gyrus (STG) and depicted a positive association with behavioral performance, that is, the higher the FC during the modality‐incompatible dual task compared to the modality‐compatible dual task, the better the performance during the modality‐incompatible dual task, *r*(45) = 0.30. Interestingly, the IFS region overlaps with the dual‐task‐related cluster and the STG with the auditory cluster. This correlation might indicate a strong interaction between a frontal control region and a sensory auditory region that might guide the successful performance of the modality‐incompatible dual task, assumed to involve modality‐based crosstalk. The second significant connection evolved between the right superior frontal gyrus (SFG), which overlaps again with the dual‐task‐related cluster, and the right superior parietal lobule (SPL), which overlaps with the parietal part of the visual cluster. Similar to the IFS‐STG connection, we found a positive correlation for the SFG‐SPL connection, *r*(45) = 0.30. Note, however, the difference that absolute mean functional connectivity was significantly higher during the modality‐compatible dual task compared to the modality‐incompatible dual task (light connection colors in Figure 8A).

**FIGURE 9:**
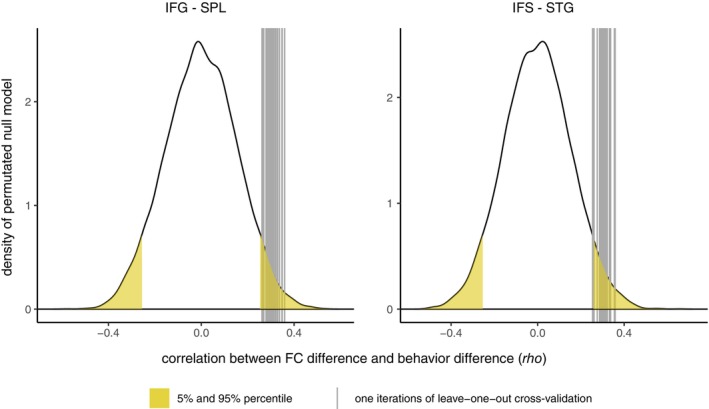
Distribution of null model and leave‐one‐out cross‐validation results. The distribution is based on a permutated null model with 10,000 iterations, with the behavioral data being permutated. The yellow areas indicate the 5% and 95% percentiles of the specific connection. The *x*‐axis depicts the range of the rho‐values for the partial correlation between FC difference and behavioral difference measure, corrected for age, sex and framewise displacement. Each of the gray lines indicates the resulting rho‐value from the leave‐one‐out cross‐validation approach. Connections were only included if on all iterations of the cross‐fold process the FC difference was different from zero (${\mathrm{BF}}_{10}>3$).

The leave‐one‐out cross‐validation approach confirmed the importance of two of the identified functional brain connections: the connection between the IFG and the SPL and the connection between the IFS and the STG. See Figure 9 for a comparison between the *rho*‐values generated by the leave‐one‐out cross‐validation and the null distribution of the *rho*‐values, specific for each connection.

We also repeated the analysis (including cross‐validation) using the Schaefer100 parcellation. The results obtained with the Schaefer100 and Schaefer200 parcellations overlapped in the connection between the IFG node (or SFG) and SPL node, a finding confirmed by cross‐validation and further supporting the relevance of these regions (compare Figures S4–S6). However, we did not find the connection between IFS and STG nodes in the Schaefer100 parcellation, which might be due to the difference in resolution between the two parcellations. For a further explanation see Figure S7, which depicts the overlap between the two parcellations and the relevant nodes. Considering the differences between parcellations, future studies should include a more fine‐grained parcellation to further confirm the relevance of these connections.

Overall, these unregistered post hoc analyses revealed significant differences in the level of individual functional brain connections related to dual‐task behavior between the two dual‐task pairings. These were observed in regions commonly involved in multitasking scenarios and modality‐based crosstalk (see Figure 8B for anatomical overlap between Schaefer regions and activity clusters).

## 4 Discussion

While multitasking is omnipresent in everyday life, little is known about the neural basis of modality‐based multitasking costs. Previous neuroimaging studies focused on univariate ROI analysis (Stelzel et al. 2006) and multivoxel pattern analysis (MVPA) (Mueckstein et al. 2025). We complement this research on modality‐based crosstalk by adopting a network neuroscience perspective. Specifically, we investigated whether the single‐task dissimilarity of whole‐brain functional connectivity differs between modality pairings, similar to the overlapping task representations revealed by the MVPA (Mueckstein et al. 2025). Additionally, we examined whether whole‐brain connectivity strength during dual‐task processing supports modality‐dependent control network involvement. Considering the robust differences in behavioral performances between modality‐compatible and modality‐incompatible pairings in dual tasks, it was surprising that we did not find a significant difference between single‐task FC dissimilarity nor different involvement of any specific network between the modality pairings during dual tasks. However, unregistered post hoc analyses comparing the FC dissimilarity of modality pairings further revealed low FC dissimilarity for the single tasks but high FC dissimilarity for the dual tasks, indicating that the dual task matrices between modality mappings differ more substantially compared to the two single‐task matrices. As preregistered whole‐brain and network comparisons between dual tasks did not reveal any differences, we additionally examined where the difference between dual‐task FCs comes from, focusing on a more local connectivity level and predefined task‐related clusters. Multiple functional brain connections differed significantly between the two modality pairings during dual‐task performance, explaining the high FC dissimilarity compared to the single tasks. Interestingly, this unregistered analysis provides preliminary evidence for significantly higher local connectivity between frontal control and sensory regions in the auditory cortex. Individual differences in the strength of this connection were associated with behavioral differences between modality‐incompatible and modality‐compatible dual‐task performance. This finding sheds light on the interplay between modality‐based crosstalk in sensory regions and the recruitment of cognitive control to resolve it, consistent with predictions from cognitive theories of multitasking (Frings et al. 2020; Janczyk et al. 2014; Koch 2009; Logan and Gordon 2001).

The fact that we did not find any whole‐brain differences between modality pairings based on FC strength, FC network dissimilarity, FC network modularity, or FC global efficiency is surprising, as many other studies reported robust differences in global network parameters for different cognitive demands. For example, Cohen and D'Esposito (2016) revealed significantly lower modularity and higher global efficiency of FC for an n‐back task compared to a resting state, indicating that the transition between rest and task is accompanied by a reconfiguration of functional connectivity (Alavash et al. 2019; Cohen and D'Esposito 2016; Cole et al. 2019; Finc et al. 2020). Similar effects were observed for the comparison between two working memory tasks (0‐back vs. 2/3‐back), which suggests that increased cognitive demand results in a decrease in network modularity (Braun et al. 2015; Finc et al. 2017, 2020; Gallen et al. 2023; Kaposzta et al. 2021; Krienen et al. 2014; Liang et al. 2016). This reduced segregation (or increased integration) of the functional brain network is reflected in dissimilarity measures (such as cosine distance) (Alexander‐Bloch et al. 2013). However, our results indicate no evidence for global reorganization between modality pairings. Thus, the data suggest that the modality pairings seem not to differ in their working‐memory load as different n‐back variations do. In contrast, despite the robust behavioral differences, the differences between modality pairings on the neural level are rather subtle and thus, whole‐brain or even network‐level analysis might be too global to detect them. This rationale is further supported by our finding of widespread network reconfiguration between single and dual tasks, showing that FC network dissimilarity can differentiate between cognitive states. One prominent difference between the cited working‐memory literature and our study design is that we also manipulated the perceptual load from single task (only one stimulus present) to dual task (two stimuli present) and not only the internal cognitive load (i.e., concurrent maintenance of two stimulus–response mappings). However, univariate fMRI studies demonstrated a relation between brain activity and the number of stimulus–response mapping rules including a manipulation of the perceptual load (Erickson et al. 2005; Marois et al. 2006; Schumacher et al. 2003). Additionally, previous studies reported that large‐scale functional brain connectivity patterns apart from the attention networks are also influenced by attentional load, for example, in the context of a multiple object tracking task (Alnæs et al. 2015). As both approaches, univariate and connectivity‐based, indicated a dependency between perceptual and cognitive load, future studies may orthogonally manipulate both types of load in a neural network approach to dissect load‐specific patterns further. The assumption of subtle rather than global neural differences between modality pairings is further supported by previous research. A study using univariate, group‐level analyses did not reveal significant dual‐task‐related differences between modality pairings at the whole‐brain level but did identify such differences exclusively within individually defined ROIs (Stelzel et al. 2006).

The challenge in this line of research is to understand the underlying neural mechanisms responsible for the robust behavioral difference between the modality pairings in dual tasks. Considering our and previous null results, instead of expecting different mechanisms, it might also be possible that both dual‐task pairings are simply processed in the same way on a neural level, resulting in different behavioral outcomes. Employing this neural processing mode for the modality‐compatible dual‐task works well but fails (i.e., produces higher behavioral dual‐task costs) when facing the modality‐incompatible pairing with additional modality‐based crosstalk. These brain processes need to be reconfigured to meet the demands of the modality‐incompatible pairing, which may then lead to performance improvement. The first hint that this reconfiguration process might require some practice is provided by the pre‐post comparison of the current data applying MPVA (Mueckstein et al. 2025). In that paper, we demonstrated that the change in single‐task representational overlap correlated with the behavioral improvement of performance after a highly specific practice intervention, specifically for the participants practicing the modality‐incompatible pairing. Future studies should extend this practice approach to a larger sample to account for individual differences and focus on dual‐task reconfiguration to strengthen this hypothesis. However, in other domains, there is already evidence for such a practice‐related network reconfiguration, for example, for motor skills (Bassett et al. 2015) and working memory (Finc et al. 2020).

Since we did not obtain significant global differences between modality pairings, we focused in our analyses on differences in functional connectivity between the two dual‐task conditions on a local connectivity level. Our findings on differences in the strength of individual functional brain connections and their relation to dual‐task performance align with results of previous dual‐task studies (Stelzel et al. 2009) and specific modality‐related research (Mueckstein et al. 2025; Stelzel et al. 2006). Note, however, that these latter analyses were not pre‐registered and should be replicated based on a priori hypotheses in future studies. Despite the required caution in interpreting our unregistered findings, we deem their report important in this regard.

The first of the two functional connections that differed significantly in strength between the modality‐compatible and the modality‐incompatible pairing links the left inferior frontal sulcus (IFS) with the right superior temporal gyrus (STG). These brain regions overlap with our task‐based activity clusters of the dual‐single task contrast (IFS), associated with cognitive control (Diveica et al. 2023; Worringer et al. 2019) and the auditory‐vocal cluster (STG), related to auditory processing (Figure 8B). The correlation with behavioral performance indicates better performance for the modality‐incompatible pairing for individuals with higher FC during the modality‐incompatible dual task compared to the modality‐compatible dual task. The involvement of the auditory regions is in line with our results from applying MVPA, which provided evidence that the single‐task representations overlap more for the modality‐incompatible pairings, specifically in the auditory regions before the practice intervention (Mueckstein et al. 2025). Generally, this temporal region seems sensitive to auditory feedback (Heinks‐Maldonado et al. 2005; Tourville et al. 2008), which further strengthens our assumption of modality‐based crosstalk being most prominent in the auditory regions due to the overlap between the auditory stimulus in one task and the expected auditory action‐effect of vocal responses in the other task. Overall, the localization of this connection, as well as its relation to behavioral performance might suggest that auditory regions need to be regulated by frontal control regions to resolve the modality‐based crosstalk and thus improve performance successfully.

The second significantly different connection links the right inferior frontal gyrus (IFG) with the right superior parietal lobule (SPL). While the IFG overlaps again with our dual‐single‐task cluster and is commonly activated in dual‐task and task‐switching studies (Worringer et al. 2019), the SPL region overlaps with the parietal part of our visual cluster. This region was identified to be essential for response‐code conflict (Paas Oliveros et al. 2023) and was demonstrated to disrupt control conflict on the perceptual level in a TMS study (Soutschek et al. 2013). In summary, both regions are often associated with different kinds of conflict requiring cognitive control. The correlation suggests that for successful behavioral performance in the modality‐incompatible pairing, this functional connection needs to be strengthened during the modality‐incompatible dual task compared to the modality‐compatible dual task. Even though the identified connections could be confirmed with a leave‐one‐out cross‐validation approach and align with previous dual‐task studies and the underlying theory, given the unregistered nature of these analyses, further studies should replicate those findings in another sample.

Cognitive theories of multitasking propose that overlapping task representations increase between‐task crosstalk, increase dual‐task costs, and require cognitive control (Frings et al. 2020; Janczyk et al. 2014; Koch 2009; Logan and Gordon 2001). This overlap in task representation is not only relevant between stimuli and responses but also at the level of the anticipated effect of responses and their modality (Greenwald 1970). Over a series of experiments (Schacherer and Hazeltine 2020, 2021, 2023) provided compelling behavioral evidence for the importance of crosstalk between stimulus modality in one task and the anticipated action effect of the other task (modality‐based crosstalk). Our neural data, analyzed with MVPA, further support these conclusions by providing evidence that overlap between neural single‐task representations in auditory regions contributes essentially to the dual‐task costs.

## 5 Conclusion

The current study complements this line of research by adopting a network neuroscience perspective and by revealing that global brain reconfiguration processes are not triggered by differences in modality compatibility. Further unregistered post hoc analyses demonstrated that during dual‐task performance, the connection between auditory regions and frontal control regions is indicative of behavioral performance. This provides potentially additional evidence for the emergence of modality‐based crosstalk. Neural network differences between modality pairings are subtle and local, undetected by global dissimilarity or network measures. Overall, our study reveals that robust and large behavioral differences are not necessarily related to global neural effects in functional brain connectivity but might be related to local differences in individual functional brain connections.

## Funding

This work was supported by Deutsche Forschungsgemeinschaft (GR 3997/4‐2, HE 7464/1‐2, RA 1047/4‐2, STE 2226/4‐2).

## Supporting information


**Data S1:** hbm70434‐sup‐0001‐Supinfo.pdf.

## Data Availability

We uploaded the BIDS transformed data (not preprocessed) to OpenNeuro: https://doi.org/10.18112/openneuro.ds005038.v1.0.1 while the beta‐images, which were used to define the group‐based activity clusters, are available on Neurovault: https://identifiers.org/neurovault.collection:16842. All scripts, stimulus material, and behavioral data can be accessed on OSF: https://osf.io/w9hsu/.
