# Supplementary material for: Network Neuroscience of Human Multitasking: Local Connections Matter
Source: Hum Brain Mapp. 2025 Dec 15;46(18):e70434. doi: 10.1002/hbm.70434 (PMC12703553; doi:10.1002/hbm.70434)
Supplement: Supplementary file 1 — Data S1: hbm70434‐sup‐0001‐Supinfo.pdf. [file HBM-46-e70434-s001.pdf]

Network Neuroscience of Human Multitasking: Local Connections Matter

Marie Mueckstein[1,2], Kirsten Hilger[3], Stephan Heinzel[4,5], Urs Granacher[2,6], Michael A. Rapp[2], and & Christine Stelzel[1]

[1] International Psychoanalytic University
Berlin
Germany
[2] Universität Potsdam
Germany
[3] Universität Würzburg
Germany
[4] Freie Universität
Berlin
Germany
[5] TU Dortmund University
Dortmund
Germany
[6] University of Freiburg
Freiburg i.B.
Germany

Author Note

Correspondence concerning this article should be addressed to Marie Mueckstein, International Psychoanalytic University, Stromstrasse 3B, 10555 Berlin. E-mail: mariemueckstein@gmail.com

Network Neuroscience of Human Multitasking: Local Connections Matter

Supplement material

Table S1

Sample size and exclusion reasons per analysis

	Excluded participants	Exclusion reason
Behavioral analysis (N=61)	10	error rate
Localizer task (N = 53)	10	error rate
	7	response-modality error
	1	head movement
Connectivity single task (N=64)	7	head movement
Connectivity dual task (N=47)	10	error rate
	14	head movement
Connectivity single vs. dual task (N=45)	10	error rate dual task
	14	head movement dual task
	2	head movement single task

Showing numbers of excluded participants.

Note the sample of the ‘Connectivity single task’ analysis was used as well for the decoding analysis at Pre timepoint reported in Mueckstein et al. (2025). In the decoding analysis, five more participants could be included as the head-movement criteria were less restrictive for the MVPA compared to the connectivity analysis.

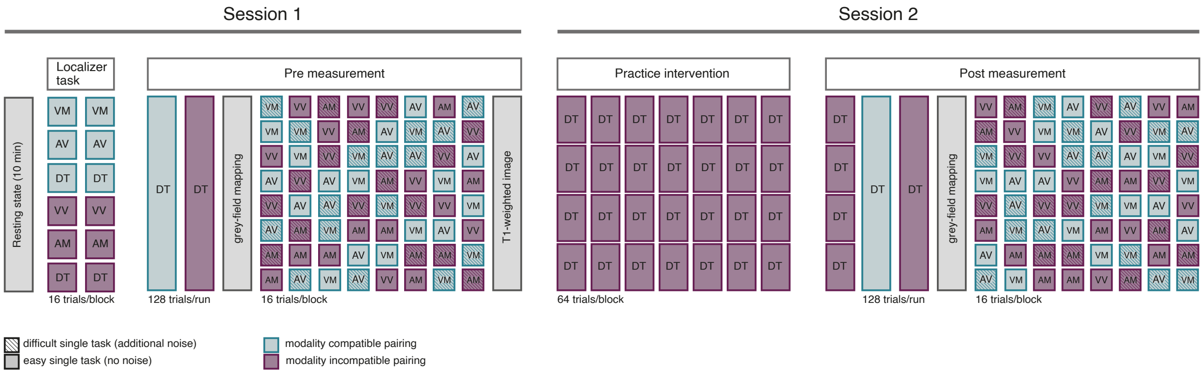

Figure S1

Structure of the fMRI Pre/Post session with a block design. The order of blocks in the Localizer Task was fixed to single task, dual task per modality mapping. The modality of the first task (visual or auditory) and the order of the modality pairing was balanced across participants. The order of the two dual-task runs in the pre session was balanced across participants as well. The following eight runs contained only single-task runs. Each run contained two blocks for the four different single tasks, one per task difficulty level, in randomized order. The practice intervention was completed outside the scanner on a computer screen, depending on the group-assignment participants either worked on modality-compatible or modality-incompatible dual tasks. VM = visual-manual, AV = auditory-vocal, VV = visual-vocal, AM = auditory-vocal, DT = dual task

Alternative FC dissimilarity parameter

Table S2

Bayes paired t-test between FC dissimilarity measured as correlation and euclidean distance of the single task modalitiy pairings

	network	Bayes Factor	error
**pearson correlation**			
	control	0.153	0.000854
	default	0.164	0.000815
	dorsAtt	0.141	0.000901
between network	limbic	0.137	0.000915
	somatomotor	0.138	0.000914
	ventrAtt	0.140	0.000903
	visual	0.229	0.000654
	control	0.237	0.000638
	default	0.451	0.000409
	dorsAtt	0.137	0.000915
within network	limbic	0.156	0.000841
	somatomotor	0.140	0.000903
	ventrAtt	0.251	0.000614
	visual	0.469	0.000398
**euclidean distance**			
	control	0.140	0.000905
	default	0.216	0.000680
	dorsAtt	0.174	0.000784
between network	limbic	0.178	0.000773
	somatomotor	0.145	0.000885
	ventrAtt	0.172	0.000791
	visual	0.153	0.000854
	control	0.203	0.000709
	default	0.988	0.000231
	dorsAtt	0.137	0.000916
within network	limbic	0.158	0.000834
	somatomotor	0.201	0.000713
	ventrAtt	0.138	0.000912
	visual	0.142	0.000897

Task-activity regressed out, schaefer200 parcellation

Functional connectivity: relative Values

In addition to the absolute connectivity values, which neglect the information if regions are positively or negatively correlated, we repeated the analysis with the relative connectivity values.

Table S3

Bayes paired t-test between relative functional connectivity of dual task modalitiy pairings

	network	Bayes Factor	error
between network	control	0.197	0.000576
	default	0.287	0.000477
	dorsAtt	0.161	0.000633
	limbic	0.168	0.000621
	somatomotor	0.170	0.000617
	ventrAtt	0.244	0.000518
	visual	0.248	0.000513
within network	control	0.184	0.000595
	default	0.302	0.000464
	dorsAtt	0.179	0.000602
	limbic	0.166	0.000624
	somatomotor	0.163	0.000629
	ventrAtt	0.217	0.000549
	visual	0.320	0.000450

Task-activity regressed out, schaefer200 parcellation

Brain-Behavior correlation

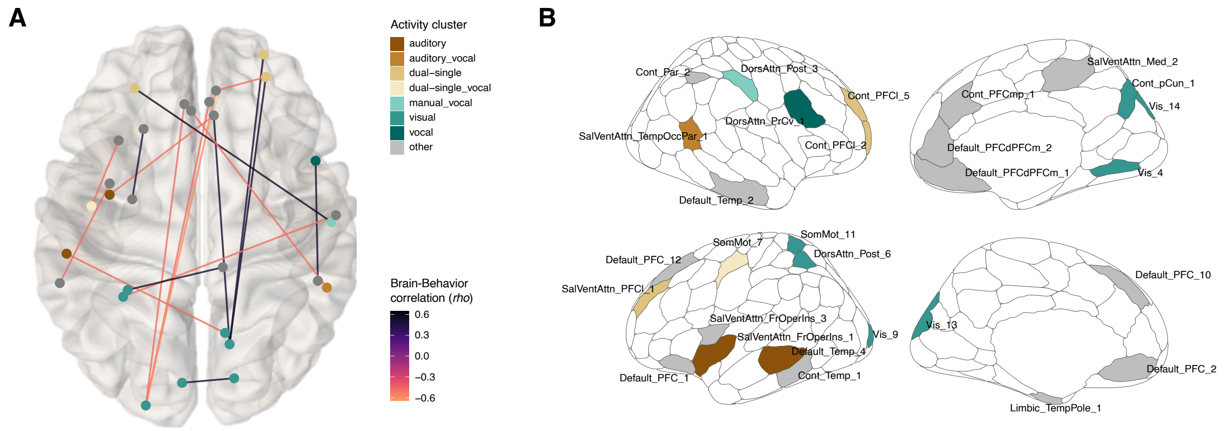

Figure S2

Relation between dual-task behavior and functional connectivity. ***A****: The superior view of the brain with the connections which are significantly related to the dual-task behavior (correlated with* $p < .001$ *and without the restriction of the region overlapping with task-relevant cluster). Color of regions indicate the corresponding activity cluster, color of connections the strengh of the correlation. The rho value is based on the partial correlation, corrected for age, gender and framewise displacement.* ***B****: Anatomical location of relevant schaefer200 regions for brain-behavior relation, together with the task-based activity clusters. Labels correspond to the Schaefer regions (name of the region according to schaefer200 7networks), colors to the activity clusters, while grey indicate no overlap between Schaefer region and our task-based activity clusters.*

Preprocessing: schaefer100 parcellation

We additionally ruled out that the results are specific to one parcellation, by repeating all the analysis with 100, instead of 200 regions, defined by Schaefer et al. (2018). All other preprocessing steps were the same as reported in the manuscript. We report the results of the Bayes paired t-test between modality pairing for the FC dissimilarity during single task, the global FC during dual task and both unregistered post-hoc analyses.

Table S4

Bayes paired t-test between modalitiy pairings for cosine distance between single task and global functional connectivity between dual-task (schaefer100 parcellation)

	network	Bayes Factor	error
**cosine distance (ST)**			
	control	0.139	0.000907
	default	0.146	0.000878
	dorsAtt	0.249	0.000618
between network	limbic	0.604	0.000332
	somatomotor	0.140	0.000904
	ventrAtt	0.203	0.000709
	visual	0.539	0.000360
	control	0.378	0.000464
	default	0.137	0.000915
	dorsAtt	0.144	0.000889
within network	limbic	0.140	0.000905
	somatomotor	0.234	0.000645
	ventrAtt	0.247	0.000621
	visual	0.137	0.000916
**global FC (DT)**			
	control	0.162	0.000632
	default	0.165	0.000626
	dorsAtt	0.196	0.000577
between network	limbic	0.161	0.000633
	somatomotor	0.183	0.000596
	ventrAtt	0.193	0.000581
	visual	0.165	0.000626
	control	0.333	0.000441
	default	0.246	0.000516
	dorsAtt	0.177	0.000606
within network	limbic	0.165	0.000626
	somatomotor	0.180	0.000601
	ventrAtt	0.314	0.000455
	visual	0.368	0.000418

Task-activity regressed out

Unregistered post-hoc: significant differences between single and dual task

Table S5

Bayes paired t-test between FC dissimilarity (cosine distance) of single and dual tasks

	network	Bayes Factor	error
	control	1.61e+34	0
	default	2.30e+35	0
	dorsAtt	1.62e+33	0
between network	limbic	7.71e+32	0
	somatomotor	5.14e+31	0
	ventrAtt	1.86e+30	0
	visual	1.04e+36	0
	control	8.42e+22	0
	default	1.62e+25	0
	dorsAtt	8.02e+15	0
within network	limbic	6.23e+11	0
	somatomotor	3.26e+17	0
	ventrAtt	3.09e+17	0
	visual	3.45e+18	0

Task-activity regressed out, schaefer100 parcellation

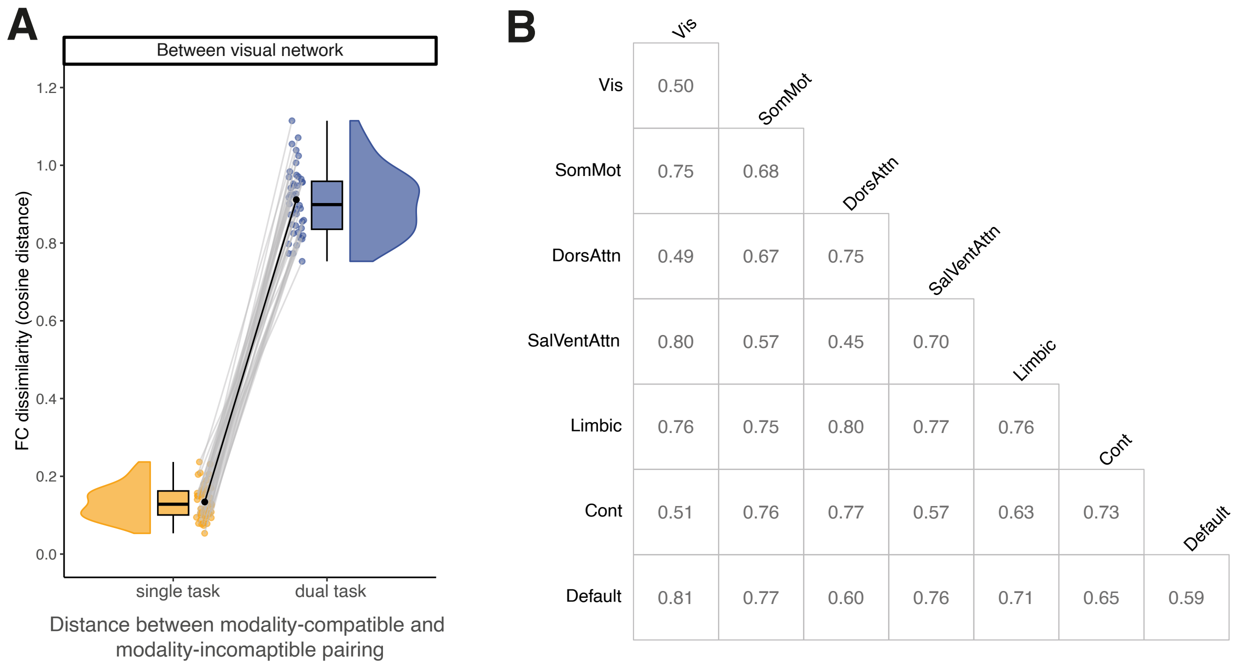

Figure S3

FC dissimilarity between modality pairings for single and dual tasks (schaefer100). ***A****: FC dissimilarity between modality pairing between the visual network and all other networks per task type. The graph provides distribution, boxplot, individual data and the mean for each task type. We identified significant difference in FC dissimilarity (cosine distance) of modality pairings between single and dual task.* ***B****: Matrix demonstrates the difference single and dual task for the cosine distance between modality-compatible and modality-incompatible for each network combination. The diagonale of the matrix describes the within network difference. We identified significant difference for all of the network combinations.*

Characterizing the difference between dual-task functional connectivity

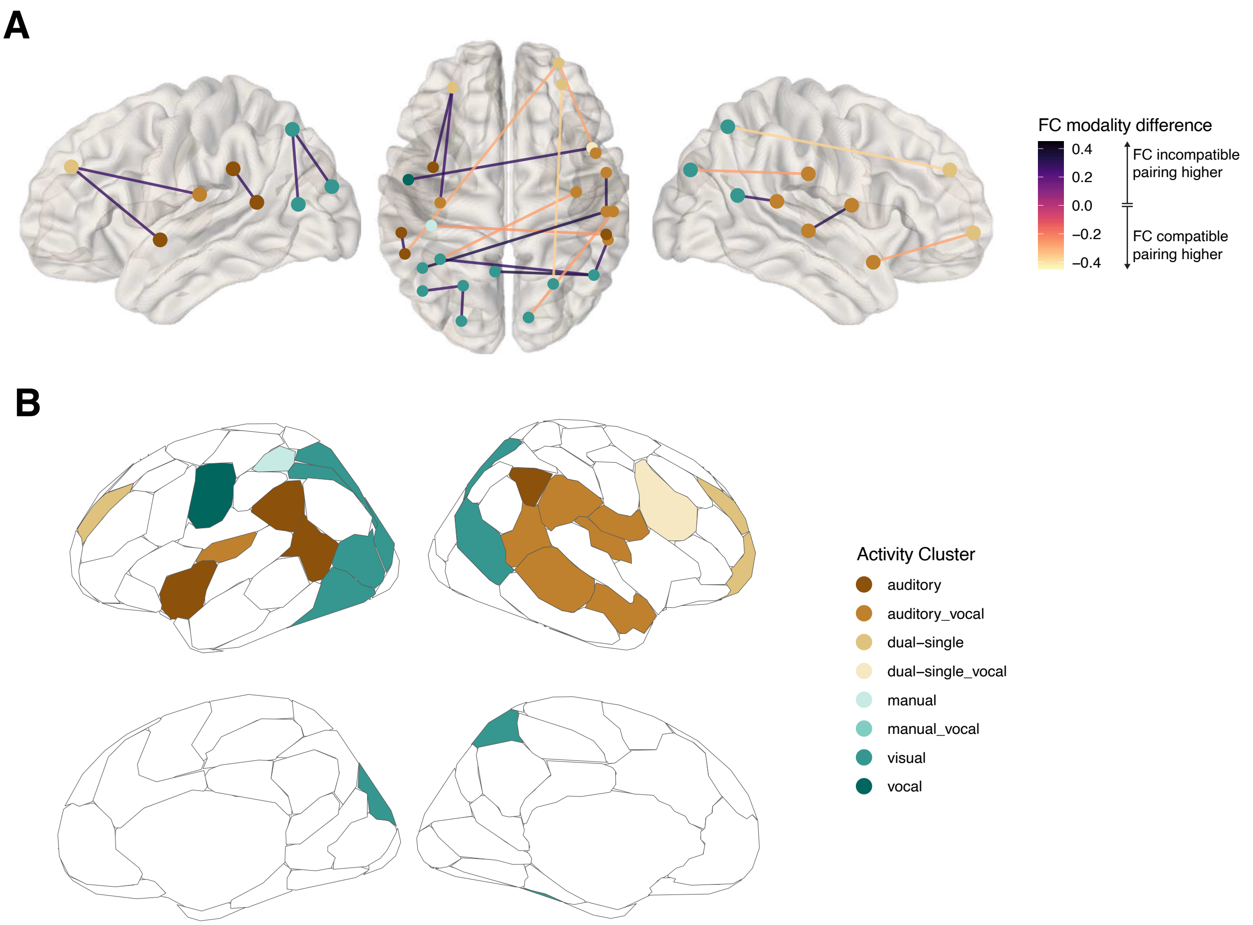

Figure S4

Significant different connections between modality pairings during dual tasks (schaefer100). ***A****: The graph depicts the connections from schaefer100 which are significantly different between the dual-task modalities, restricted to regions overlapping with task-related activity clusters. Color of regions indicate the corresponding activity cluster, color of connections the difference value of functional connectivity (FC) between modality-incompatible dual task (positive values) and modality-compatible dual task (negative values). The two sagital views contain only regions and connections within the corresponding hemisphere. The superior view contains all significant connections.* ***B****: The graph depicts the schaefer100 parcellation in medial and sagital view. Color of the regions indicate the corresponding task-related activity clusters with which the region overlaps. White regions did not overlap with any of our activity clusters.*

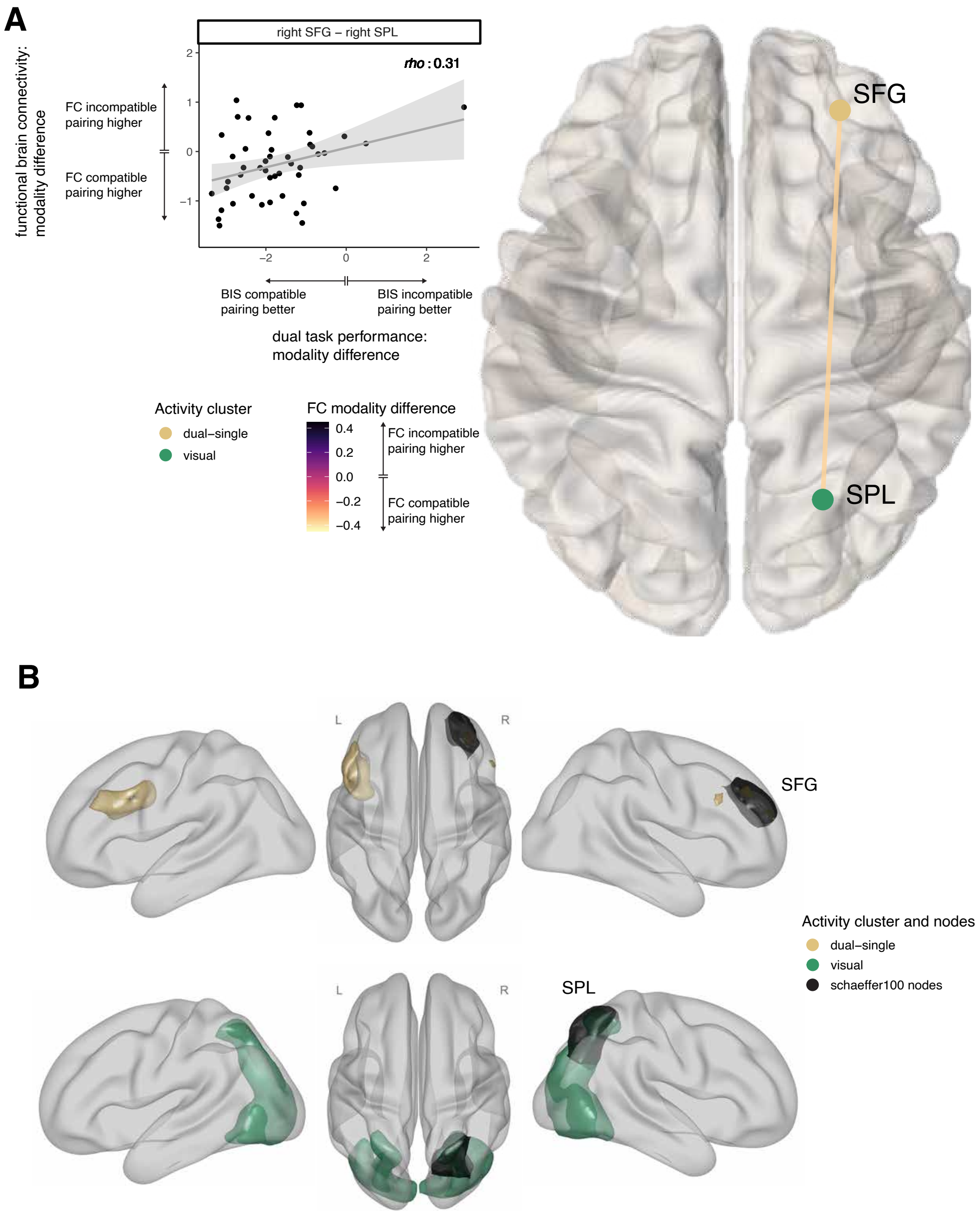

Figure S5

Relation between dual-task behavior and functional connectivity (schaefer100).

A: *The superior view of the brain with the connection significantly different between dual-task modalities and significantly related to the dual-task behavior based on the schaefer100 parcellation and validated by a leave-one-out cross-validation). Color of regions indicate the corresponding activity cluster, color of connections the difference value of functional connectivity (FC) between modality-incompatible dual task (positive values) and modality-compatible dual task (negative values). The scatter plot corresponds to the brain connection and depicts each individual as one point, whereas the y-axis represents the difference score between modalities of the functional connectivity during dual tasks and the x-axis the difference score between modalities of the behavioral performance during dual task, operationalized as BIS parameter. The corresponding rho-value is based on the partial correlation, corrected for age, gender and framewise displacement.* ***B***: *Anatomical location of relevant schaefer100 regions for brain-behavior relation, together with the task-based activity clusters. Label abbreviations: SFG = superior frontal gyurs, SPL = superior parietal lobule*

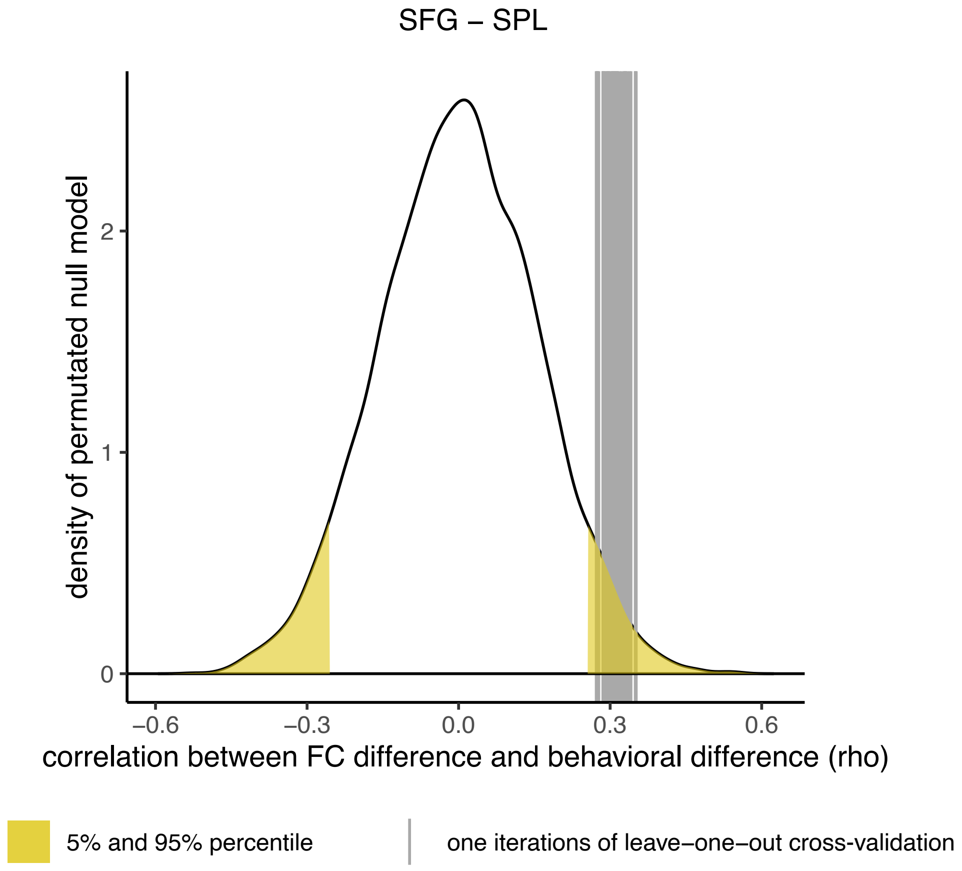

Figure S6

Distribution of null model and leave-one-out cross-validation results (schaefer100).

The distributation is based on a permutated null model with 10,000 iterations, with the behavioral data being permutated. The yellow area indicate the 5 % and 95 % percentile of the specific connections based on the schaefer100 parcellation. X-axis depicted the range of the correlation value rho for the partial correlation between FC difference and behavioral difference measures, corrected for age, sex and framewise displacement. Each of the grey lines indicates the resulting rho-value from the leave-one-out cross-validation approach. Connections were only included if on all iterations of the crossfold process the FC difference was different from zero ($BF_{10} > 3$*).*

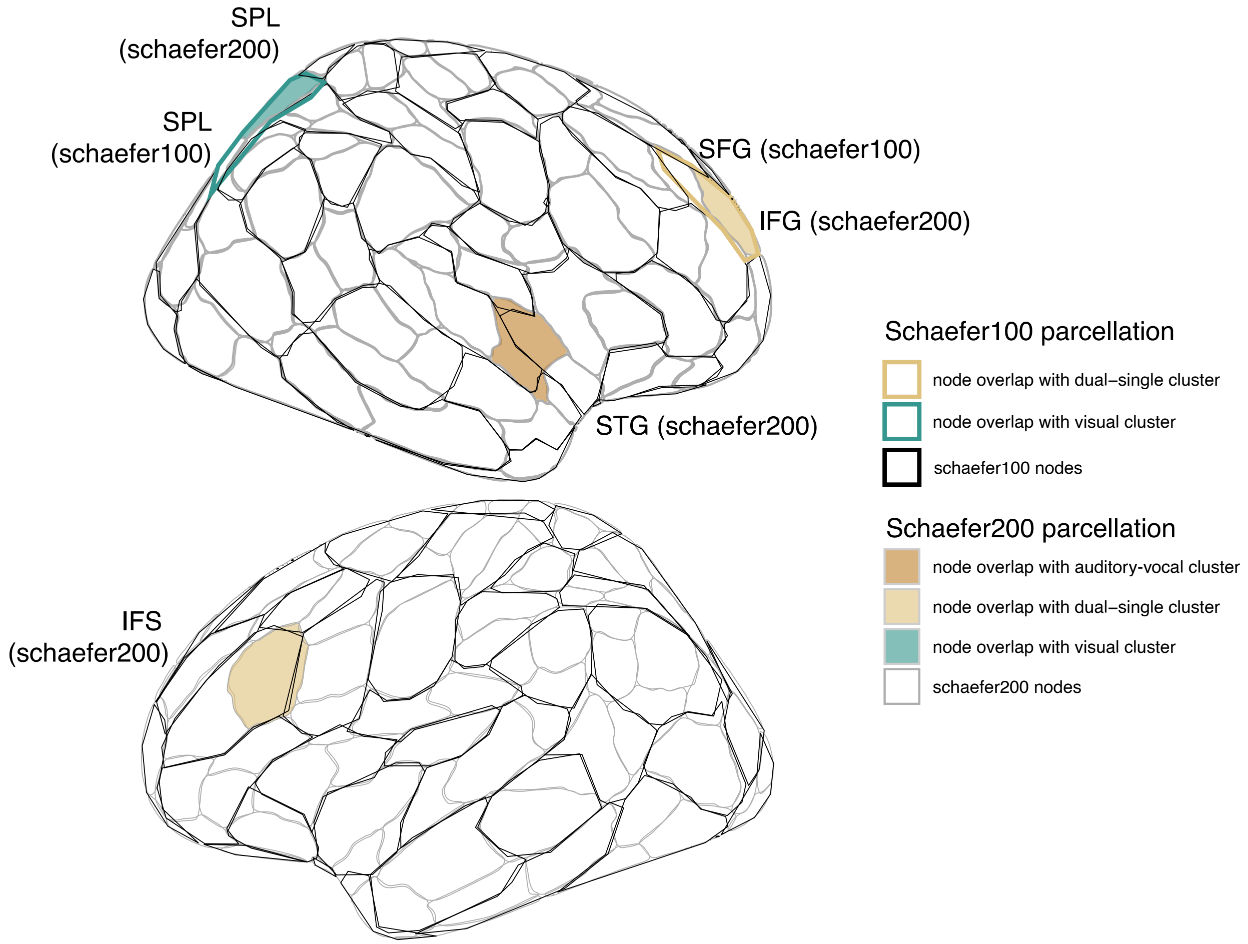

Figure S7

Overlap of all regions between schaefer100 and schaefer200 parcellation. Color of regions indicate the corresponding activity cluster only for those regions that remain after the selection and correlation process. Regions with a colored border belong to the schaefer100 parcellation, regions filled with color to the schaefer200 parcellation. Note, the STG node in the schaefer200 overlaps partly with three different nodes from the schaefer100 parcellation. The IFS node becomes two times bigger in the schaefer100, which comes with increased variance. Both observed differences, the overlap with several other nodes and an increase in size, might explain why the correlation and/or the FC between modality pairings differ and thus the results between schaefer200 and schaefer100 only partly overlap.

Preprocessing: neural-task activity remained

To test the robustness of our findings, we ruled out that the non-significant results are due to the regression of task-related activity in the preprocessing step. Accordingly, we repeated the pre-registered analysis with timeseries data where neural-task activity is still remaining. All the other preprocessing steps were the same as reported in the manuscript. We report the results of the Bayes paired t-test between modality pairing for theFC dissimilarity during single task and the global FC during dual task.

Table S6

Bayes paired t-test between modalitiy pairings for cosine similarty between single task and global functional connectivity between dual-task (task-related activity remained)

	network	Bayes Factor	error
**cosine distance (ST)**			
	control	0.168	0.000802
	default	0.140	0.000906
	dorsAtt	0.404	0.000443
between network	limbic	0.137	0.000916
	somatomotor	0.187	0.000748
	ventrAtt	0.140	0.000906
	visual	0.139	0.000910
	control	0.535	0.000362
	default	0.187	0.000749
	dorsAtt	4.932	0.000000
within network	limbic	0.137	0.000915
	somatomotor	0.147	0.000878
	ventrAtt	0.139	0.000907
	visual	0.137	0.000915
**global FC (DT)**			
	control	0.164	0.000628
	default	0.310	0.000458
	dorsAtt	0.229	0.000535
between network	limbic	0.222	0.000543
	somatomotor	0.484	0.000358
	ventrAtt	0.428	0.000384
	visual	0.162	0.000632
	control	0.180	0.000602
	default	0.160	0.000635
	dorsAtt	0.558	0.000330
within network	limbic	0.271	0.000491
	somatomotor	0.158	0.000638
	ventrAtt	0.251	0.000511
	visual	0.164	0.000627

schaefer200 parcellation

Graph parameters

A common approach in network analysis are the use of graph parameters to describe the network based on graph theory. We calculated two measures,the network modularity, which describes how well the network can be subdivided into non-overlapping parts and the global efficiency, which quantifies the efficiency of communication in a network as the average of inverse shortest path length (Rubinov & Sporns, 2010). Scripts to calculate both parameters can be found on OSF https://osf.io/w9hsu/.

Global modularity

We applied the same algorithm as Hilger and Fiebach (2019) which is based on the Louvain algorithm (Blondel et al., 2008). We used a fixed gamma of 1 and repeated the Louvain community detection 100 times to maximize the modularity parameter Q. We selected the 'negative_asym' option to again account for negative connections in the matrices, which is suggested by Rubinov and Sporns (2010) in the Brain Connectivity toolbox. We calculated the modularity per participant for single, and dual task and for each modality pairing (total of four matrices). Input was the fishers z-transformed functional connectivity matrices. Similar to the other analysis, we were interested in the effect of modality pairing, testing it with a linear-mixed effect model, while controlling for head movement, age, gender and number of runs (formula per analysis option: $globalEfficiency \sim task * mapping + meanFD + run + age + sex$) and calculated and report the Bayes Factor for a paired t-test between modality pairing for single and dual tasks (Table S7).

Table S7

Bayes paired t-test between modalitiy pairings of global modularity score for each combination of task (single and dual task) with gamma = 1

	Bayes Factor	error
DT	0.158	0.000638
ST	0.138	0.000913

Task-activity regressed out, schaefer100 parcellation

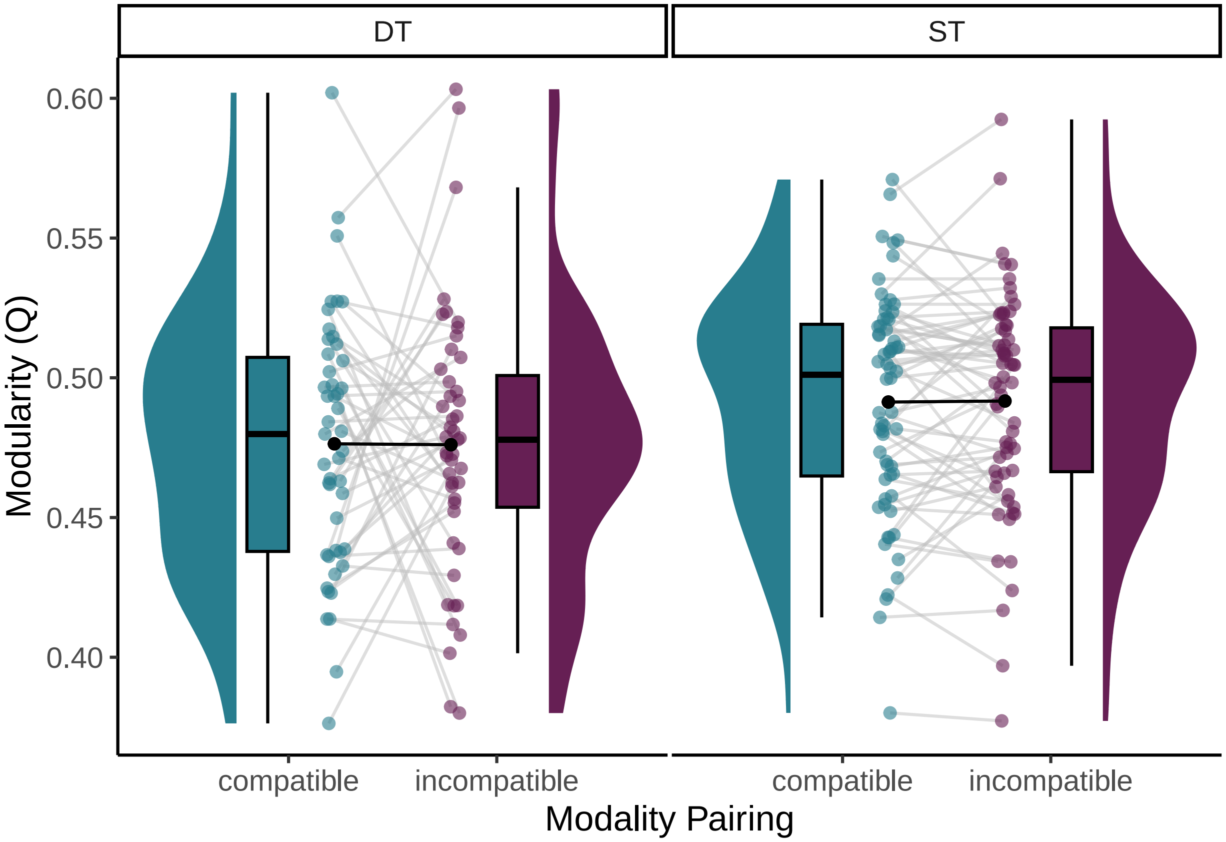

Figure S8

Global modularity per task type comparing modality pairings. Graph shows distribution, boxplot, indidvidual data (color points) and mean value per modality pairing (black). DT = dual task, ST = single task, gamma = 1.

Similar to the other results, we did not find any evidence for a different modularity between modality pairings (see Figure S8) and the Baysian paired t-test (Table S7). In sum, we did not find any evidence that the neural network is differently structured in communities for the modality-compatible pairing as for the modality-incompatible pairing.

Global efficiency

We used the function *efficiency_wei* from the Brain connectivity Toolbox (Rubinov & Sporns, 2010) and applied three different forms of handling negative connections in the FC matrices: filter, absolute and rescale. In the filter option we set the negative connections to NaN, in the absolute option we take only absolute connections and the last option is to rescale all value to the interval [0,1]. As there is no agreement of the best practice how to include negative connections, we applied all three options and compared the results. Input to all three options are the task-related 200x200 fisher's z-transformed FC matrices per participant, separately for single, dual task and for both modality pairings (total of four matrices). The same mixed model and Bayes paired t-test was used as for the global modularity.

Table S8

Bayes paired t-test between modalitiy pairings of global efficiency score per negative-weight treatment

	options	Bayes Factor	error
DT	abs	0.263	0.000499
	filt	0.548	0.000334
	resc	0.223	0.000542
ST	abs	0.509	0.000376
	filt	0.416	0.000433
	resc	0.137	0.000916

Task-activity regressed out, schaefer200 parcellation

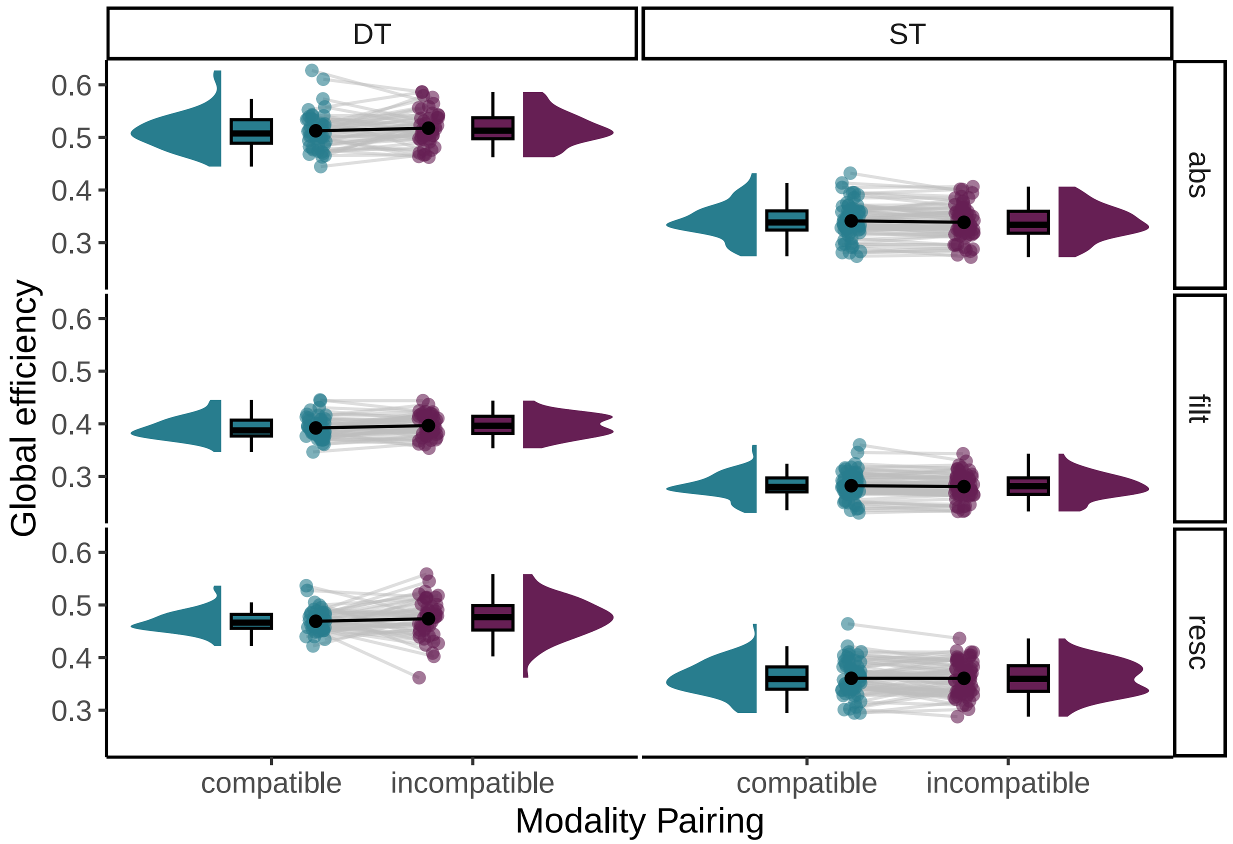

Figure S9

Global efficiency per task type and analysis option comparing modality pairings. Graph shows distribution, boxplot, indidvidual data (color points) and mean value per modality pairing (black). DT = dual task, ST = single task, abs = absolute FC matrix, filt = filtered for only positive weigths, resc = rescale FC matrix to interval [0,1].

In sum, we did not find evidence for differences in how efficient the network communicate between the modality-compatible and modality-incompatible pairing, neither during single, nor during dual task (compare Figure S9 and the Bayes Factor in Table S8).

References

Blondel, V. D., Guillaume, J.-L., Lambiotte, R., & Lefebvre, E. (2008). Fast unfolding of communities in large networks. *Journal of Statistical Mechanics: Theory and Experiment*, *2008*(10), P10008. https://doi.org/10.1088/1742-5468/2008/10/P10008

Hilger, K., & Fiebach, C. J. (2019). ADHD symptoms are associated with the modular structure of intrinsic brain networks in a representative sample of healthy adults. *Network Neuroscience*, *3*(2), 567–588. https://doi.org/10.1162/netn_a_00083

Mueckstein, M., Görgen, K., Heinzel, S., Granacher, U., Rapp, M. A., & Stelzel, C. (2025). Multitasking Practice Eliminates Modality-Based Interference by Separating Task Representations in Sensory Brain Regions. *The Journal of Neuroscience*, *45*(4), e0755242024. https://doi.org/10.1523/JNEUROSCI.0755-24.2024

Rubinov, M., & Sporns, O. (2010). Complex network measures of brain connectivity: Uses and interpretations. *NeuroImage*, *52*(3), 1059–1069. https://doi.org/10.1016/j.neuroimage.2009.10.003

Schaefer, A., Kong, R., Gordon, E. M., Laumann, T. O., Zuo, X.-N., Holmes, A. J., Eickhoff, S. B., & Yeo, B. T. T. (2018). Local-Global Parcellation of the Human Cerebral Cortex from Intrinsic Functional Connectivity MRI. *Cerebral Cortex*, *28*(9), 3095–3114. https://doi.org/10.1093/cercor/bhx179
